# Plexciton Dirac points and topological modes

**DOI:** 10.1038/ncomms11783

**Published:** 2016-06-09

**Authors:** Joel Yuen-Zhou, Semion K. Saikin, Tony Zhu, Mehmet C. Onbasli, Caroline A. Ross, Vladimir Bulovic, Marc A. Baldo

**Affiliations:** 1 Department of Chemistry and Biochemistry, University of California–San Diego, La Jolla, California 92093, USA; 2 Department of Chemistry and Chemical Biology, Harvard University, Cambridge, Massachusetts 02138, USA; 3 Department of Physics, Kazan Federal University, Kazan 420008, Russian Federation; 4 Department of Physics, Massachusetts Institute of Technology, Cambridge, Massachusetts 02139, USA; 5 Center for Excitonics, Research Laboratory of Electronics, Massachusetts Institute of Technology, Cambridge, Massachusetts 02139, USA; 6 Department of Materials Science and Engineering, Massachusetts Institute of Technology, Cambridge, Massachusetts 02139, USA; 7 Department of Electrical Engineering and Computer Science, Massachusetts Institute of Technology, Cambridge, Massachusetts 02139, USA

## Abstract

Plexcitons are polaritonic modes that result from the strong coupling between excitons and plasmons. Here, we consider plexcitons emerging from the interaction of excitons in an organic molecular layer with surface plasmons in a metallic film. We predict the emergence of Dirac cones in the two-dimensional band-structure of plexcitons due to the inherent alignment of the excitonic transitions in the organic layer. An external magnetic field opens a gap between the Dirac cones if the plexciton system is interfaced with a magneto-optical layer. The resulting energy gap becomes populated with topologically protected one-way modes, which travel at the interface of this plexcitonic system. Our theoretical proposal suggests that plexcitons are a convenient and simple platform for the exploration of exotic phases of matter and for the control of energy flow at the nanoscale.

## Abstract (web summary)

Ultraviolet-visible molecular excited states (excitons) may couple to collective excitations in metals (plasmons) to form plexitons, which transfers energy ballistically over tens of microns. Here, the authors propose a plexitonic system which exhibits Dirac points and topologically nontrivial band structure.

## Graphical abstract

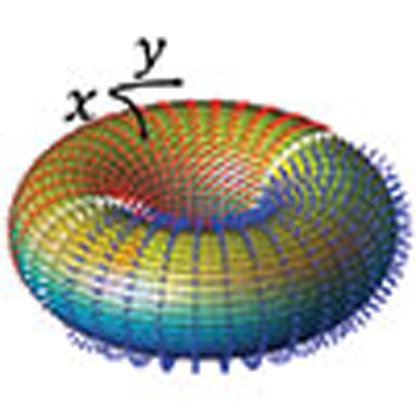

When ultraviolet–visible light is absorbed by an organic molecular aggregate, it promotes molecules from their ground states to their excited electronic states. The resulting excitations, known as excitons, can migrate between molecules via a mixture of coherent and incoherent processes^1^. Understanding and controlling how this migration of energy occurs is a fundamental problem of chemistry and physics of condensed phases. Furthermore, it is also a technological problem which is relevant to the development of efficient organic solar cells and light-emitting devices as well as all-optical circuitry^2^. Many strategies to control the motion of excitons exist, a particularly interesting one being where they couple to surface plasmons (SPs)^3^. In such strategy, the spatial coherence of plasmons assists the transport of an exciton across length-scales that are orders of magnitude larger than regular exciton diffusion lengths. When the coupling is strong, meaning that the energy exchange between the exciton and plasmon is faster than the respective decay times^4^,^5^,^6^,^7^,^8^, plexcitons (a class of polaritons) emerge^9^,^10^ and energy can migrate ballistically over the coherence length of the plasmon (between 10 and 40 μm)^11^. Besides their importance for energy transport, organic plexcitons promise to be an exciting room-temperature laboratory for the study of light-matter and many-body interactions at the nanoscale^8^.

On the other hand, topologically nontrivial states of matter have been a topic of great interest in condensed matter physics owing to the discoveries of the quantum Hall effect^12^, and more recently, of topological insulators^13^,^14^. The systems supporting these states are characterized by topological invariants^15^, integer numbers that remain unchanged by weak perturbations. Physically, a nontrivial topological invariant signals the presence of one-way edge modes that are immune against moderate amount of disorder. Even though these phenomena were first conceptualized for fermions in solids, they have been successfully generalized to bosonic systems including photons in waveguides^16^,^17^,^18^, ring resonator arrays^19^, ultracold atoms in optical lattices^20^ and classical electric circuits^21^,^22^. Furthermore, we have recently proposed an excitonic system consisting of a two-dimensional porphyrin film, which becomes topologically nontrivial in the presence of a magnetic field^23^. A challenging feature of that proposal is the requirement of large magnetic fields (⩾10 T) and cryogenic temperatures to preserve exciton coherence.

In this work, we consider a conceptually different platform, which, by recreating Dirac cones and topologically protected edge modes in plexcitons, avoids the use of large magnetic fields and, under appropriate circumstances, may work at room temperature. In the last year, Dirac and topological polaritons have been proposed in other contexts, such as optomechanical arrays and inorganic materials in optical cavities. All of these works share a common goal to ours, which is the design of exotic modes in strongly coupled light-matter systems. However, there are substantial qualitative and quantitative differences arising from the choices of material (organic exciton versus inorganic exciton^24^,^25^,^26^ or mechanical mode^27^,^28^) and electromagnetic (SP versus microcavity^24^,^25^,^26^ or photonic crystal^27^,^28^) excitations. Hence, the physics involved in our plexciton system contrasts with the other proposals in terms of the energy and length-scales involved in the excitations, the magnitude of the couplings, the generation of nontrivial topology and the experimental conditions for its realization. Organic excitons differ from their inorganic counterparts in that they have large binding energies and are associated with large transition dipole moments. In general, SP electromagnetic fields are strongly confined compared with those in microcavities because of the hybridization of light with charge oscillations in the metal^29^. The combination of all these properties in the organic plexciton context gives rise to strong light-matter interactions even at room temperature and in an open cavity^8^. We believe that, by introducing topological band theory concepts into the realm of plexciton systems, the present article yields a fresh perspective to the degree of control of energy transport which is achievable in the nano- and mesoscales.

## Results

### Plexciton Dirac points

**Figure 1:**
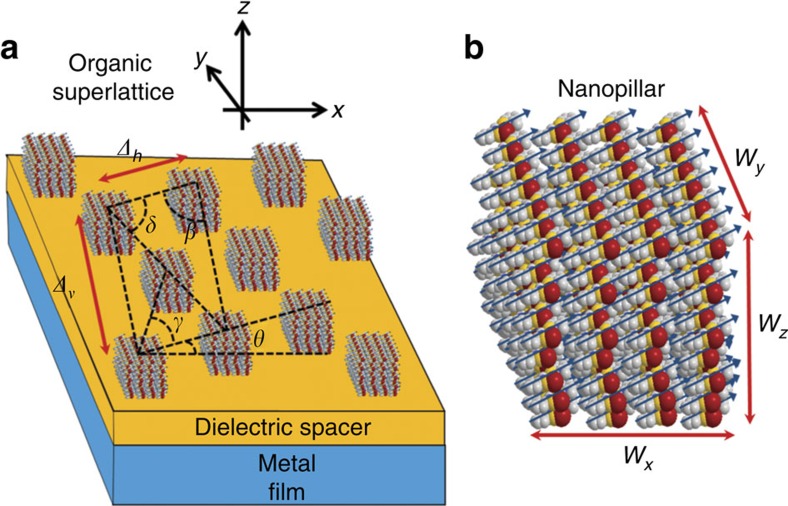
Plexciton setup. (**a**) It consists of a plasmonic metal film, a dielectric spacer and an organic layer. The latter is taken to be a monoclinic superlattice of organic nanopillars, which makes an angle *θ* with respect to the *x* axis and is further characterized by angles *β*, *γ*, *δ* as well as distances between nanopillars Δ_*h*_ and Δ_*v*_. When the density of emitters in the organic nanopillars is big enough, the coupling between the excitons in the organic layer and the surface plasmons (SPs) in the metal becomes larger than their linewidths, giving rise to polaritonic eigenmodes that are superpositions of excitons and plasmons, or more succintly, plexcitons. In this article, we shall also consider the case where the dielectric spacer is a magneto-optical (MO) material. The superlattice design is not essential for our purposes, except for guaranteeing a global gap for topological modes spanning all wavevectors. Hence, one may substitute it with a standard molecular crystal with some trade-offs. (**b**) Zoom-in view of a nanopillar. It is a parallelepiped of dimensions *W*_*i*_ along each axis, and consists of closely packed organic emitters (represented by balls and sticks) each featuring a transition dipole (blue arrows).

The setup of interest is depicted in Fig. 1. It consists of three layers: a plasmonic metal modelled with a Drude permittivity 
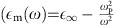
, with constants 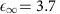, *ω*_P_=8.8 eV, which are representative parameters for Ag), an *a*=80 nm thick dielectric spacer (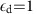) and an organic layer (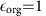). The spacer is placed to avoid quenching of organic excitons by single-particle excitations in the metal upon close contact^30^. Later on, we will consider the case when this dielectric spacer is also endowed with magneto-optical (MO) properties. For purposes of band-structure engineering, we take the organic layer to be an oblique superlattice of organic nanopillars (Fig. 1a) with unit cell angles *β*, *γ*, *δ* and an angle *θ* with respect to the *x* axis of the setup (in the absence of this superlattice, a standard molecular crystal may be used with some trade-offs, as explained below). The nanopillars are rectangular parallelepipeds of densely packed organic chromophores (assuming a van der Waals distance between chromophores of 0.3 nm, *ρ*_np_=38 chromophores per nm^3^) with volume *V*_np_=*W*_*x*_*W*_*y*_*W*_*z*_ (Fig. 1b), obtained from growing a J-aggregate film^31^,^32^. J-aggregation of chromophores results in a collective transition dipole **μ**_**n**_ for the **n**-th nanopillar. Dipolar interactions between the collective dipoles couple the various nanopillars. Assuming perfect periodicity of the superlattice and only nearest and next-nearest neighbour (NN and NNN) dipolar interactions, we can write the Hamiltonian for excitations in the plexciton setup as *H*=∑_**k**_*H*_**k**_ (see the Methods for details),


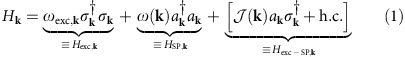


where,


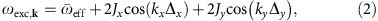



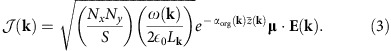


Here, *ω*_exc,**k**_ and *ω*(**k**) are dispersions of the exciton and plasmon, respectively. *J*_*i*_ and Δ_*i*_ are effective NN hoppings and spacings between nanopillars along the *i*-th axis, respectively, 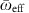 is the effective nanopillar site energy, and we have taken **μ**_**n**_=**μ** for all **n**. Equation (3) denotes the **k**-dependent coupling between a plasmonic mode 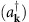 and a collective exciton state 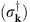 throughout the organic layer. It is proportional to the square root of the total effective number of nanopillars 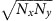 coherently coupled to each SP mode^33^,^34^,^35^ (*N*_i_ is the number of nanopillars along the *i*th axis) and to the collective nanopillar transition dipole moment **μ**, which scales as 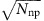, where *N*_np_ is the number of chromophores per nanopillar (see the Methods, Modelling of superlattice). Overall, 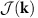 scales as 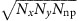, the total number of chromophores in the superlattice, as expected from a plexciton problem^7^. *S* and *L*_**k**_ denote the SP mode surface area and mode length, respectively, and *ɛ*_0_ is the permittivity of vacuum.

**Figure 2:**
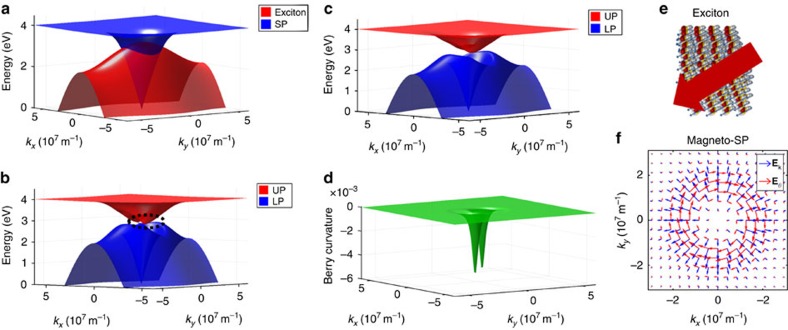
Bulk plexciton properties. Dispersion relations: (**a**) for SP and exciton (organic layer) modes independently, (**b**) when they couple in the absence of the MO effect, yielding lower (LP) and upper (UP) plexciton branches, which feature two Dirac cones (dashed black circle), and (**c**) when they couple in the presence of the MO effect (*g*=0.3), lifting the Dirac cones. (**d**) (unnormalized) Berry curvature associated with the LP in **c**. Physical mechanism for the appearance of plexciton Dirac points: (**e**) we specialize to nanopillar transitions, which are parallel to the *xy* plane and (**f**) plot of magnitude of the electric field of magneto-SP modes as a function of wavevector. In the absence of the MO effect, only the wavevector-parallel components **E**_**k**_(blue) are present. Thus, the nanopillars experience no coupling with modes whose wavevectors are perpendicular to the transition dipole. Along these directions, degeneracies between the SP and the exciton modes are not lifted, yielding two plexciton Dirac points. Nonzero tangential components **E**_***θ***_ (red) emerge upon inclusion of the MO effect, lifting these degeneracies.

Figure 2a shows superimposed dispersion curves for *H*_exc,**k**_ and *H*_SP,**k**_ independently (setting *H*_exc−SP_=0 in the calculations, see the Methods for parameters). We assume that **μ** is parallel to the *xy* plane, and design our superlattice to obtain *J*_*x*_, *J*_*y*_>0, yielding a dome-like dispersion for *H*_exc,**k**_; that is, it features a maximum at **k**=0, behaving as a two-dimensional (2D) H-aggregate^1^. As for *H*_SP,**k**_, its (time-reversal symmetric) dispersion has the shape of a rotationally symmetric fountain, and is nothing more than the 2D rendering of the standard one-dimensional textbook result^36^, featuring a linear dependence of the energy at short wavevectors and a plateau at large ones, indicating excitations that are qualitatively closer to light or to charge oscillations in the metal, respectively (Supplementary Notes 1 and 2, as well as Supplementary Figs 1–4). Figure 2b shows the two-plexciton-branch band-structure arising from the diagonalization of equation (1). We notice that anticrossing gaps are opened in the vicinity of where the dispersion curves for *H*_exc,**k**_ and *H*_SP,**k**_ used to cross in Fig. 2a. This is a signature of exciton-SP coupling *H*_exc,SP,**k**_. Given a fixed wavevector direction, whenever these anticrossings occur, the lower-plexciton (LP) branch starts off as being mostly SP at short **k** values, but parametrically morphs into mostly exciton at large ones; the opposite happens with the upper-plexciton (UP) branch. However, the most striking feature of Fig. 2b is the appearance of two Dirac cones (see dashed circles) at critical wavevectors k* where anticrossings do not happen. Their onset coincides with the directions at which **k** is orthogonal to **μ**. Their physical origin is explained in Fig. 2e, which, in its top panel, shows that the in-plane electric field for the **k**-th SP mode, **E**_⊥_(**k**)≡**E**(**k**)−**E**(**k**)·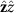, is purely parallel to **k**, **E**_⊥_(**k**)=**E**_**k**_(**k**) (blue vector field). If all the dipoles in the organic layer are aligned (in-plane) along **μ**, their projection onto the SP electric field, which gives rise to the exciton-SP coupling (see equation (3) as well as Supplementary Note 3 and Supplementary Figs 5–8), will wax and wane as a function of the azimuthal angle 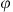 between the fixed dipole and the varying SP wavevector according to **μ**·**E**(**k**)∝cos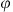. Clearly, this projection will vanish if **k** happens to be orthogonal to **μ**, that is, at the special angles 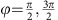, so that any degeneracy between the exciton and the SP modes will remain unlifted along these directions. From this physical picture, we can extract the two essential ingredients for the emergence of the plexciton Dirac cones. First, the dipoles need to be aligned to create an anisotropic 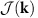 as a function of 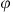. Second, this alignment needs to be horizontal, as a vertical component of the dipole will couple to the vertical component of the electric field 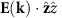, and this coupling, unlike its horizontal counterpart, does not vanish for any 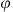. It is important to note that neither of these properties require the use of the superlattice, which will be exploited for a different purpose (the design of a global gap for topologically protected edge modes, as explained in the next paragraphs). Therefore, a standard organic molecular crystal with aligned transition dipoles lying on the horizontal *xy* plane will suffice. These plexciton Dirac cones, which, to our knowledge, have not been reported in the past, should be easily detectable by collecting the reflected light spectra upon excitation of the plexcitonic system in a grating, Otto or Kretschmann configurations^36^, by systematically scanning across |**k**| and 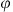 values. For a general (|**k**|, 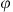), the spectrum should consist of two dips as a function of dispersed energy, each associated with the corresponding eigenenergies of the LP and UP. However, the two dips merge at the Dirac cones. In a standard plexciton dispersion measurement, one only scans across |**k**|. As we are interested in a two-dimensional dispersion, the scan must also be performed across 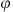. To trace the formation of the Dirac points, Supplementary Note 3 and Supplementary Figs 9–11 show two-dimensional cross-sections of the dispersion curves in Fig. 2.

### Plexciton topological modes

Having elucidated the mechanism for the formation of plexciton Dirac cones, we proceed to entertain a more ambitious goal. We aim to engineer topologically protected plexcitons by opening the Dirac cones using a time-reversal symmetry breaking (TRSB) perturbation^15^. To accomplish this, we now assume that the dielectric spacer has MO properties; that is, upon application of a perpendicular magnetic field, its permittivity becomes anisotropic, 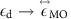,


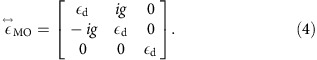


Materials associated with this dielectric tensor exhibit Faraday effect and are of great interest in the fabrication of optical isolators^37^. For the near-infrared and visible spectra, yttrium iron garnets (Y_3_Fe_5_O_12_, YIG) substituted with Bi (BiYIG), Ce (CeYIG) or other rare earths provide high Faraday rotation with low optical absorption^38^,^39^. In this article, we are interested in the new SP modes, denoted as magneto-SP modes, arising at the interface of the plasmonic metal and the MO layer. The solution for the latter is highly non-trivial, and we refer the reader to our solution in the Supplementary Notes 1 and 2, which build a perturbation theory on the small MO parameter *g*, generalizing a simpler calculation of Chiu and Quinn^40^. Figure 2c shows the same calculation as Fig. 2b except for the inclusion of the MO effect. Interestingly, we notice that the Dirac cones have been lifted, yielding a global gap because of the H-aggregate superlattice dispersion. A physical understanding of the latter phenomenon can be obtained by appealing to Fig. 2e again. Within our perturbation theory, the magneto-SP modes differ from their original SP counterparts in that there are additional tangential components (**E**_*θ*_, red) to the electric field. The clockwise vortex vector field is a signature of TRSB; it becomes counterclockwise upon change of direction of the magnetic field. This tangential electric field is solely responsible for opening the Dirac cones at the critical angles 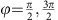, where the original field (blue) ceased to couple to the excitons. Hence, we have concocted a situation where anticrossings occur for all azimuthal angles 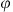. To characterize the topology of the resulting band-structure, we numerically compute the Berry curvature for each plexciton branch^41^; we show that of the LP in Fig. 2d. Its integral with respect to the Brillouin zone is the so-called Chern number *C*, an integer which, if nonzero, signals a topologically nontrivial phase. Figure 2d clearly shows that this integral is non-vanishing, and in fact, adds up to *C*=−1 (by the sum rule of Chern numbers, the upper branch necessarily has *C*=1). Intuitively, it is also clear that most of the nontrivial topology, and hence, Berry curvature, is concentrated in the vicinity of what used to be the Dirac cones. In passing, we note that considerable attention has recently been given to magneto-SPs, where the magnetic field is applied parallel (instead of perpendicular) to the metal film itself, yielding dispersion relations, which are nonreciprocal^40^,^42^. Curiously, this arrangement does not give us the vortex vector field we are looking for, although it might be intriguing to explore the connection between these magneto-SPs and the ones exploited in our present work, arising from a perpendicular magnetic field.

**Figure 3:**
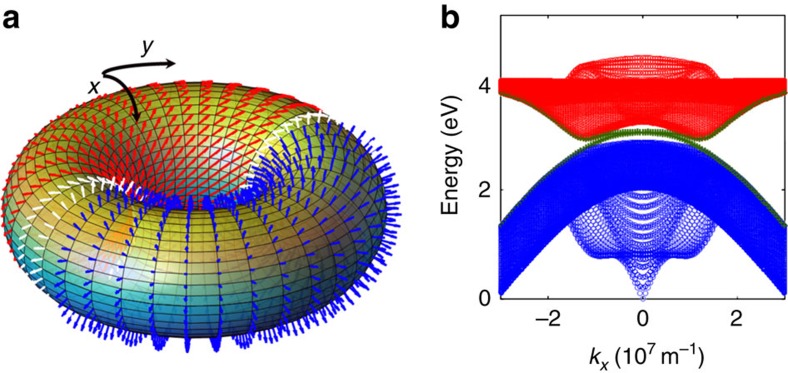
Topologically protected edge modes. (**a**) Simulation of edge modes where magneto-SPs are computed in the torus geometry. Two domains of organic layers are placed on top of it (just like two icings on a donut). In-plane (red) and out-of-plane (blue) transition dipoles yield topologically nontrivial and trivial plexcitons, respectively. Topologically protected one-way plexcitons appear at the interfaces (white domains). Each interface features a different plexciton direction of motion. (**b**) One-dimensional dispersion relation *ω*(*k*_*x*_) for the setup in **a**. Bulk LPs (blue) and UPs (red) separated by edge modes (green) featuring positive and negative dispersions, respectively, and localized along each interface.

So far, all the described calculations have been carried out in the bulk. By virtue of the bulk-boundary correspondence^15^, we expect topologically protected one-way edge modes associated with this setup. To compute them, it is convenient to keep periodic boundary conditions for the magneto-SP modes, yet consider two domains of excitons on top (Fig. 3a), one with (in-plane) dipoles pointing along **μ** (red dipoles) and the other one with vertical dipoles along 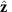 (blue dipoles). Pictorially, this setup resembles a donut with two icings, where the donut is the metal with toroidal geometry, and the two icings are the domains of excitons separated by two interfaces located at 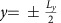 and *y*=0, where *L*_*i*_ is the total width of the simulated sample along *i* (in our calculations, we take *L*_*x*_=40 μm, *L*_*y*_=6 μm). The Chern numbers associated with the bulk LP branch of each domain are *C*=−1 and *C*=0, respectively. Hence, the plexcitons for the blue domain are topologically trivial. This can be understood by recalling that no plexciton Dirac points occur when dipoles are vertically aligned, regardless of the MO effect. As this system is perfectly periodic along the *x*-direction, *k*_*x*_ is still a good quantum number, and Fig. 3b shows the corresponding plexciton dispersion. This band-structure is essentially a projection of the gapped 2D bulk band-structures of both domains of plexcitons onto one axis *k*_*x*_ with additional states spanning the gap between the LP and UP branches. Inspection of the nature of these mid-gap states reveals that they have substantial exciton and magneto-SP character, and that they are precisely the edge states we are searching for: one band has positive (negative) dispersion and is localized along *y*=0 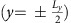. Thus, by preparing a plexciton wavepacket localized along one of the interfaces, and making sure it is composed of energy states within the global topological gap, one ensures that transport occurs robustly without much probability of backscattering. The reason being that elastic backscattering requires coupling between counterpropagating modes, which are separated by a distance 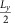, which is large compared with the width of the corresponding wavefunctions along 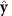.

Figure 3b was generated with the parameters 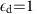, *g*=0.3, yielding a minimum gap between plexciton branches (at the wavevectors k* of the original Dirac points) of 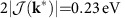. The crossing of the SP and exciton dispersion curves happens at 2.86 eV. Given typical linewidths associated with the various dissipative mechanisms at room temperature (*γ*_exc,rel_∼5 MeV, *γ*_exc,deph_∼40 MeV, *γ*_SP,rel_∼10 MeV, where rel and deph stand for relaxation and dephasing), our parameters lie within the strong-coupling regime, namely, 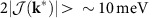 (ref. 7). Ideally, we would like to have all edge states lying within the global topological gap. For the present simulation, only a fraction of the latter (close to the anticrossings) lies within the global topological gap; the rest are degenerate with bulk plexcitons. This scenario arises as a consequence of the large exciton dispersion anisotropy (*J*_*x*_≠*J*_*y*_) compared with the strength of 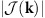 for all **k**. The latter is a result of optimization: thicker MO spacers yield larger values of 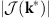 but, due to the evanescent nature of SP modes, also reduce the overall couplings 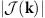 for **k**≠k* (Supplementary Note 3). Typical MO garnets have similar *g* but higher 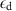 values (for an external magnetic field of 0.01 T, 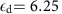 and *g*=0.1 (ref. 38)), yielding a strong index mismatch in our setup. As explained in Supplementary Note 3, our future aim is to boost 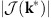 by considering various MO layer thicknesses *a* as well as novel MO garnet compositions, which maximize the 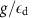 ratio, the latter of which is not fundametally limited. Strategies may include the consideration of MO garnet sphere arrays^43^, plasmonic/magnetic metal nanostructures^44^, Ce substituted YIGs^45^ or Eu nanocrystals^46^.

**Figure 4:**
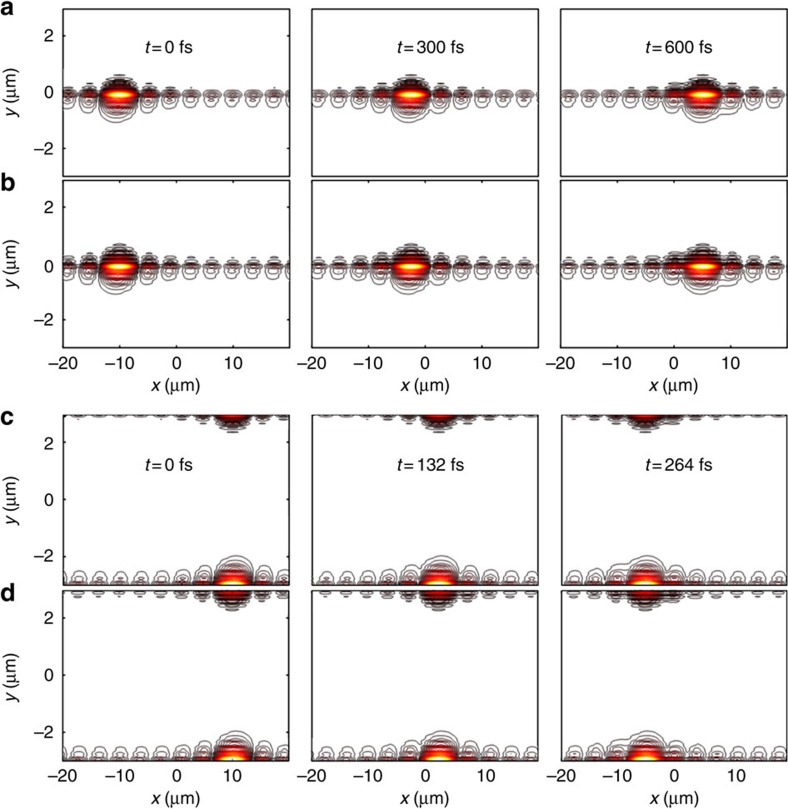
Snapshots of dynamics of topologically protected edge modes. (**a**–**d**) The dynamics of an initially localized plexciton along the *y*=0 and 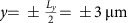 interfaces of our simulation is shown. (**a**–**d**) The exciton and magneto-SP components are shown, respectively. These modes, which are robust to disorder, have substantial excitonic and magneto-SP components and travel in opposite directions along different interfaces.

Figure 4a,b shows snapshots of the dynamics associated with the edge states lying within the global gap. Figures 4a,b and c,d show an edge plexciton wavepacket that starts localized at 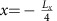 and 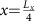, respectively, and track the one-way (to the left or to the right) nature of its micrometre-scale motion within the subpicosecond timescale. The dispersion of the edge plexcitons is such that a subset falls within the light cone 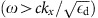 so far-field excitation and detection of this fraction is possible via direct interaction with the organic layer. The rest of the plexcitons can be probed using the already mentioned SP measurement techniques, by launching plexcitons exciting the metallic layer itself. Furthermore, the ballistic and one-way nature of these modes can in principle be demonstrated using fluorescence microscopy^47^. As explained, the robustness of one-way transport for these edge modes, even in the presence of disorder, relies on the global topological gap, which is a consequence of our 2D H-aggregate-type superlattice design (see the Methods for details). In terms of experimental feasibility, we note that some compromise on topological protection might be acceptable if using a plain molecular crystal is much easier than constructing the superlattice as long as scattering between *k*_*x*_ states is not strong.

## Discussion

To summarize, we have described the design of exotic plexcitons via a judicious choice of material and electromagnetic excitation modes. We showed that Dirac cones and topologically protected edge states emerge from relatively simple hybrid organic/inorganic nanostructures. Even though we have not precisely identified an explicit MO material which fully satisfies our requirements, we believe its design is within reach, and is the subject of our present investigations. It is also worth noting that the physical origin of the described edges states is different from that of edge plasmons in disk geometries^48^,^49^, although the connections are worth exploring. An interesting extension of this work is the consideration of plexcitonic Dirac point opening without TRSB, which might give rise to rich phases in analogy to other Dirac systems^50^. In general, the possibility of directed migration of excitation energy at the nano- and mesoscale offers exciting prospects in light-harvesting and all-optical circuit architectures. Furthermore, given the recent experimental discovery of nonlinear many-body effects such as Bose–Einstein condensation of organic cavity-polaritons^51^,^52^,^53^ and plexcitons^54^ at room temperature, the introduction of the novel features described in this letter enriches the scope of these materials as a test-bed for novel many-body quantum phenomena.

## Methods

### Hamiltonian of the plexciton setup

A quantum mechanical description of the plexciton setup is given by a Hamiltonian,


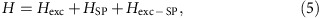


where each of the terms denotes the energetic contributions from the excitons in the organic layer, the SPs and the coupling between them. More specifically (*ħ*=1),


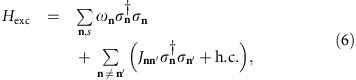



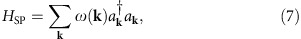



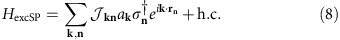


Here, 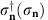 and 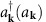 label the creation (annihilation) operators for the collective exciton at the **n**-th nanopillar and the **k**-th SP mode, respectively, where **n** and **k** are (two-dimensional) in-plane vectors denoting a position and a wavevector, respectively. J-aggregation of chromophores results in a collective transition dipole **μ**_**n**_ at an excitation energy *ω*_**n**_, whereas the dispersion energy of the **k**-th SP mode is denoted *ω*(**k**). Dipolar interactions 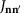 couple the various nanopillars. The coupling between the exciton and the SP depends on the average in-plane location **r**_**n**_ of the **n**-th nanopillar, and is also dipolar in nature,


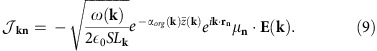


Here, *S* is the SP mode surface area, *L*_**k**_ denotes a vertical (*z*-direction) mode-length of the SP, which guarantees that the total energy of a SP prepared at the **k**th mode is quantized at the energy *ω*(**k**), **E**(**k**) is an appropriately scaled electric field of the corresponding mode, and 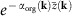 yields a mean-field average of the interaction of the evanescent SP field (with decay constant *α*_org_ in the organic layer) over the chromophores at different vertical positions of the nanopillar; it optimizes the interaction such that one may assume the nanopillar is a point-dipole located at the mean height 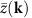. The latter average renders the originally 3D system into an effectively 2D one. Detailed derivations of equations (6)–(9), 50, 51, 55 are available in the Supplementary Notes 1–3.

Assuming perfect periodicity of the superlattice (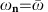, **μ**_**n**_=**μ**) and only NN and NNN dipolar interactions, we can re-express *H*_exc_ (equation (6)) in terms of **k** modes. As explained in Supplementary Note 3, it is possible to approximate *H*_exc_ up to *O*(|**k**|^2^) as arising from an *effective* simplified rectangular (rather than monoclinic) lattice aligned along the *x*, *y* axes, and with NN interactions only. This is a reasonable thing to do, as the topological effects we are interested arise at relatively long wavelengths. Within this approximation, we may construct Fourier modes 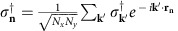 (here, *N*_*i*_ is the effective number of nanopillars along the *i*-th direction) and rewriting equation (5) in reciprocal space, we obtain 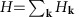, where *H*_**k**_ is given by equation (1).

### Modelling of superlattice

For our simulations, both of the band-structure and the edge states, we chose the length parameters Δ_*h*_=100, Δ_*v*_=88, *W*_*x*_=10, *W*_*y*_=75, *W*_*z*_=70 nm, and take *β*=13.1° (see Supplementary Note 3 for explanation of choice of parameters). Denoting the transition dipole 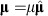, we estimate 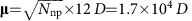 (*D*=Debye), where *N*_np_=*ρ*_np_*V*_np_ is the number of chromophores in the nanopillar, *ρ*_np_=37 chromophores per nm^3^; 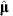 is the *in-plane* unit vector making an angle of *α*=224°) with respect to 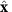, that is, 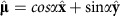. Choosing the simulation values for *H*_exc_ in equation (2) to be Δ_*x*_=Δ_*y*_=50 nm, we obtained the effective parameters 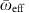
=0.57 eV, *J*_*x*_=1.04 meV and *J*_*y*_=0.31 meV. As explained in Supplementary Note 3, for strong exciton-plasmon coupling, the relevant density of chromophores is the weighed average between the density in the nanopillars and the null one in the void space. The density and vertical thickness of this superlattice are in line with the typical parameters that yield strong coupling in plexciton systems^7^.

### Experimental considerations

In terms of the fabrication of the plexciton setup, we warn that the creation of BiYIG layers typically need high temperature and oxygen, which is incompatible with deposition on Ag or organic materials. Hence, the MO layer could first be deposited on garnet substrates such as GGG (Gd_3_Ga_5_O_12_) (111), and subsequently floated off by dissolving or polishing the substrate. One should then transfer the film on a Ag-coated substrate and the organic layer may be deposited and patterned on garnet.

### Effects of disorder and global gap

It is important to note that, owing to the topological nature of these states, perfect lattices are not required, so the robustness of the one-way edge states holds as long as orientational and site energy disorder induce perturbations which are smaller than the topological anticrossings. We tested these ideas by simulating lattices with disorder in the site energies 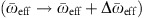 as well as in the orientations of the dipoles 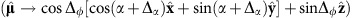, where Δ_*j*_ are chosen to be Gaussian random variables centred at 0 and having disorder widths *σ*_*j*_ for each 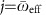, *α*, *φ*. By systematically varying these widths independently and keeping track of the presence of the one-way edge states, we noticed that the latter survive under large amounts of disorder, whose thresholds are approximately located at 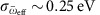, *σ*_*α*_∼57° and *σ*_*φ*_∼28°. Furthermore, we suspect that these values are lower bounds, as the disorder in these simulations is exacerbated by the torus configuration of our simulation, where periodicity along the *x* axis is conserved. In other words, owing to the latter, for a given superlattice coordinate along the *y* axis, we fixed the same values of disorder Δ_*j*_ across all *x* (that is, the disorder was perfectly correlated along *x*).

One may check that if *J*_*x*_≤0 or *J*_*y*_≤0, the global topological gap is not guaranteed anymore, and edge modes may become degenerate with bulk modes. Under certain circumstances, these two types of modes could hybridize because of impurity potentials, yielding channels connecting one edge to the other, opening backscattering channels. Importantly, however, the resulting band-structure would formally remain topologically nontrivial (in terms of the Chern numbers *C*≠0 for the plexciton bands) even in the absence of such global gap, so even if perfect one-way transport is not observed in these cases, signatures of the latter may remain. The latter observation applies if it is experimentally more convenient to use of a standard molecular crystal (with negligible exciton dispersion) rather than the proposed superlattice. These issues will be explored in future work. In the mean time, it suffices to note that, as a proof of concept, a global gap that hosts topologically protected edge states can be obtained by using an H-aggregate-type organic superlattice like the one suggested in this article.

## Additional information

**How to cite this article:** Yuen-Zhou, J. *et al*. Plexciton Dirac points and topological modes. *Nat. Commun.* 7:11783 doi: 10.1038/ncomms11783 (2016).

## Supplementary Material

Supplementary Information Supplementary Figures 1-11, Supplementary Notes 1-3 and Supplementary References
