# Supplementary material for: Plexciton Dirac points and topological modes
Source: Nat Commun. 2016 Jun 9;7:11783. doi: 10.1038/ncomms11783 (PMC4906226; doi:10.1038/ncomms11783)
Supplement: Supplementary Information — Supplementary Figures 1-11, Supplementary Notes 1-3 and Supplementary References [file ncomms11783-s1.pdf]

Supplementary Note 1. MAGNETO-SPS AT A METAL-DIELECTRIC INTERFACE (TWO-LAYER SETUP)

A. Maxwell's equations

Maxwell's equations in arbitrary media read as follows,

$$\nabla \cdot \vec{D} = 0, \tag{1}$$
$$\nabla \cdot \vec{B} = 0, \tag{2}$$
$$\nabla \times \vec{E} = -\partial_t \vec{B}, \tag{3}$$
$$\nabla \times \vec{H} = \partial_t \vec{D}. \tag{4}$$

We are interested in the case where the electric displacement $\vec{D}$ and the magnetic field $\vec{H}$ are related to the corresponding electric fields $\vec{E}$ and the magnetic inductions $\vec{B}$ via the linear constitutive relations $\vec{D} = \epsilon_0 \overleftrightarrow{\epsilon}\vec{E}$ and $\vec{H} = \mu_0^{-1}\overleftrightarrow{\mu}^{-1}\vec{B}$, where $\epsilon_0 = 8.854 \times 10^{-12}\frac{\mathrm{C}}{\mathrm{Vm}}$ and $\mu_0 = 1.257 \times 10^{-6}\frac{\mathrm{kgm}}{\mathrm{C}^2}$ are the permittivity and permeability of vacuum, and $\overleftrightarrow{\epsilon}$ and $\overleftrightarrow{\mu}$ are the corresponding scaling (unitless) tensors for the medium in question. By taking the curl of Supplementary Equation (3), we obtain the wave equation,

$$\nabla(\nabla \cdot \vec{E}) - \nabla^2 \vec{E} = -\frac{\overleftrightarrow{\epsilon}}{c^2}\partial_t^2 E, \tag{5}$$

where we have introduced the free space speed of light $c = (\mu_0\epsilon_0)^{-\frac{1}{2}}$. A completely analogous equation holds for H by taking the curl of Supplementary Equation (4), but the latter suffices for our purposes.

B. Permittivities

We are interested in (magneto-)SPs arising at the interface between a plasmonic metal (silver or gold) and a dielectric medium which is endowed with magneto-optical (MO) properties (see Supplementary Figure 1). The latter can be a mix of a magnetic oxide [1–3] dissolved in a polymer. We assume that the magnetization of both layers is null, $\overleftrightarrow{\mu} = 1$.

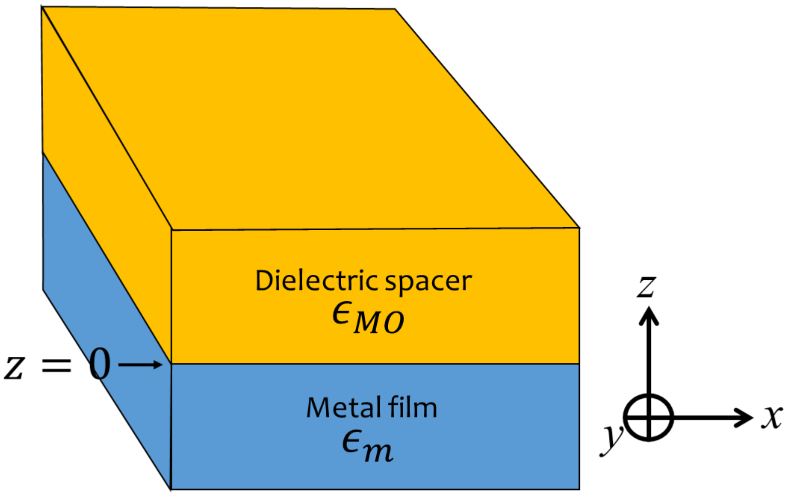

Supplementary Figure 1. *Metal-MO dielectric interface at* $z = 0$. We solve for the surface plasmon (SP) modes arising at the metal-dielectric interface, in particular, when the permittivity of the dielectric $\overleftrightarrow{\epsilon}_{MO}$ is anisotropic due to the application of a perpendicular external magnetic field. The SP modes in this case are referred to as magneto-SP modes. As a first approximation to the exciton-SP coupling, we assume that the organic layer is embedded within the dielectric spacer medium.

The permittivity of the metal is taken to be isotropic $\overleftrightarrow{\epsilon} = \epsilon_m \overleftrightarrow{I}$, where

$$\epsilon_m(\omega) = \epsilon_\infty - \frac{\omega_P^2}{\omega^2 + i\omega\gamma} \tag{6}$$

is of the Drude form (the parameters for Ag (Au) are $\epsilon_\infty = 3.7(6.9)$, $\omega_P = 8.8\,\mathrm{eV}$, and $\gamma = 0.01(0.07)\,\mathrm{eV}$ [4]). Throughout this work, we will set $\gamma = 0$ in order to keep the formalism simple. Physically, as long as the relevant energy scales of interest are larger than γ (see main text), this is a good approximation. Since we are interest in the plexciton (strong exciton-SP coupling) regime [5–11], this should be a good approximation to take. Otherwise, the quantization of the problem becomes much more complicated.

For the MO layer, the permittivity is anisotropic: upon interaction with an external magnetic field in the perpendicular z-direction, it acquires the form,

$$\overleftrightarrow{\epsilon}_{MO} = \begin{bmatrix} \epsilon_d & ig & 0 \\ -ig & \epsilon_d & 0 \\ 0 & 0 & \epsilon_d \end{bmatrix}, \tag{7}$$

where the tensor has been written in Cartesian coordinates $(\hat{\boldsymbol{x}}, \hat{\boldsymbol{y}}, \hat{\boldsymbol{z}})$, and we take $\epsilon_d = 1$ and $g = 0.1$. Here, the off-diagonal term is proportional to the Faraday rotation that a linearly polarized plane-wave electric field experiences as it passes through the material; g changes sign upon change of magnetic field direction. Typically, MO magnetic oxides like Bismuth- and Yttrium-Iron Garnets (BIG, YIG) [1, 2] have permittivities of $\epsilon_d \sim 6$ [1–3], which imply a severe index mismatch with the metal and organic layers of interest. Hence, we are implicitly assuming that we have a Maxwell garnet blend with a low index polymer or aerogel [12] at our disposition, which yields an effective $\epsilon_d = 1$. On the other hand, $g \sim 0.1$ is a reasonable parameter for MO garnets under a magnetic field of 0.1 Tesla [1]. Just as with ϵ_m, we ignore imaginary (absorptive) contributions to ϵ_d. Section B of Supplementary Note 3 discusses other MO materials that could be used for the purposes of our study.

Chiu and Quinn [13] have solved a slightly different problem, namely, the magneto-SPs arising from a metal under a strong magnetic field coupled to a isotropic non-MO dielectric. In their study, the anisotropy arises in the metal permittivity rather than in the one corresponding to the dielectric. The resulting equations are, as expected, very similar, and one could translate their equations to our setup by some careful changes of variables. However, for clarity of presentation and in order to develop the three-layer calculation of Supplementary Note 2, which is a generalization of the two-layer case, we shall outline the entire procedure here. Importantly, in doing so, we manage to go further than Chiu and Quinn and construct a perturbation theory in the small parameter g. This allows us to develop explicit expressions for the electromagnetic modes which, as far as we are aware, have not appeared in the literature before.

C. Electromagnetic modes for each layer

The problem is rotationally symmetric about the vertical z-direction, so it is convenient to adopt a cylindrical coordinate system. Let us search for SP modes labeled by $\boldsymbol{k}$ which propagate in-plane and decay along $\hat{\boldsymbol{z}}$ (this is precisely the condition for SP modes),

$$\vec{E}(\boldsymbol{k}) = \boldsymbol{E}(\boldsymbol{k}) e^{i(kr_k + k_z z - \omega t)}, \tag{8.1}$$
$$\vec{B}(\boldsymbol{k}) = \boldsymbol{B}(\boldsymbol{k}) e^{i(kr_k + k_z z - \omega t)}. \tag{8.2}$$

For a given direction of $\boldsymbol{k}$, we shall write vectors in the right-handed cylindrical coordinate system spanned by the unit vectors $\hat{\boldsymbol{k}}, \hat{\boldsymbol{\theta}}_{\boldsymbol{k}}, \hat{\boldsymbol{z}}$ such that $\hat{\boldsymbol{k}} \times \hat{\boldsymbol{\theta}}_{\boldsymbol{k}} = \hat{\boldsymbol{z}}$. for instance, $\boldsymbol{E} = (E_k, E_{\theta_k}, E_z)$, where $E_i = \boldsymbol{E} \cdot \hat{\boldsymbol{i}}$ (beware that we have defined the tangential direction of $\hat{\boldsymbol{\theta}}_{\boldsymbol{k}}$ with respect to $\hat{\boldsymbol{k}}$ and not to $\hat{\boldsymbol{r}}$). Physically, $\boldsymbol{k}$ and $\omega = \omega(\boldsymbol{k})$ denote the in-plane (propagating) wavevector and frequency of the monochromatic wave, respectively, $r_k = \boldsymbol{r} \cdot \hat{\boldsymbol{k}}$ is the projection of the position vector $\boldsymbol{r} = (r_k, r_\theta, z)$ along the $\hat{\boldsymbol{k}}$ direction, and k_z is the imaginary wavevector associated with the evanescent wave along the perpendicular direction. Inserting these modes into Supplementary Equation (5) yields an anisotropic wave equation for $\boldsymbol{E}$,

$$\sum_{ljm} [(\delta_{il}\delta_{jm} - \delta_{im}\delta_{jl}) k_j k_l + \Omega^2 \epsilon_{im}] E_m = 0, \tag{9}$$

where we have used the notation $\Omega(\boldsymbol{k}) = \frac{\omega(\boldsymbol{k})}{c}$ corresponding to the free space wavevector. Here, the i, j, l, m indices run through k, θ, z and, formally, we may write $\boldsymbol{k} = (k_k, k_\theta, k_z) = (k, 0, k_z)$. Due to rotational symmetry, $\overleftrightarrow{\epsilon}_{MO}$ has the same form in the $\hat{\boldsymbol{k}}, \hat{\boldsymbol{\theta}}_{\boldsymbol{k}}, \hat{\boldsymbol{z}}$ coordinates as Supplementary Equation (7).

1. MO layer ($z > 0$)

Inserting the dielectric tensor associated with the MO layer (Supplementary Equation (7)) into Supplementary Equation (9) yields a matrix equation $\mathbb{M}_{MO}\boldsymbol{E}_{MO} = 0$ which explicitly reads as,

$$\begin{bmatrix} \epsilon_d - \Omega^{-2}k_{z,MO}^2 & ig & \Omega^{-2}kk_{z,MO} \\ -ig & \epsilon_d - \Omega^{-2}(k^2 + k_{z,MO}^2) & 0 \\ kk_{z,MO}\Omega^{-2} & 0 & \epsilon_d - \Omega^{-2}k^2 \end{bmatrix} \begin{bmatrix} 1 \\ E_{\theta,MO} \\ E_{z,MO} \end{bmatrix} = \begin{bmatrix} 0 \\ 0 \\ 0 \end{bmatrix}. \tag{10}$$

At this point, we have chosen the arbitrary normalization condition $E_{k,MO} = 1$. The secular equation corresponding to Supplementary Equation (10) is,

$$\left(\frac{k_z}{\Omega}\right)^4 + \mathbb{B}\left(\frac{k_z}{\Omega}\right)^2 + \mathbb{C} = 0, \tag{11}$$

where,

$$\mathbb{B} = 2\left[\left(\frac{k}{\Omega}\right)^2 - \epsilon_d\right], \tag{12.1}$$

$$\mathbb{C} = \left[\left(\frac{k}{\Omega}\right)^2 - \epsilon_d\right]\left[\left(\frac{k}{\Omega}\right)^2 - \frac{\epsilon_d^2 - g^2}{\epsilon_d}\right]. \tag{12.2}$$

The bi-quadratic Supplementary Equation (11) yields solutions $k_{z,MO} = i\alpha_{MO}^{\pm}, -i\alpha_{MO}^{\pm}$, where $\alpha_{MO}^{\pm}$ are two different evanescent decay or exponentially rising constants given by,

$$\alpha_{MO}^{\pm} = \Omega\sqrt{\frac{\mathbb{B}}{2} \pm \sqrt{\frac{\mathbb{B}^2}{4} - \mathbb{C}}}. \tag{13}$$

Note that these (in general, complex-valued) constants $\alpha_{MO}^{\pm}$ must have positive real part for $e^{-\alpha_{MO}^{\pm}z}$ to decay or for $e^{\alpha_{MO}^{\pm}z}$ to rise, respectively. In the first two-layer setup we are considering, we will assume that the MO layer extends indefinitely for $z > 0$ so the field in this region must be a superposition of the two evanescent fields; exponentially rising fields will become important when we add an additional interface at $z = a$ (see Supplementary Note 2). The tangential and perpendicular components of the electric field (given $E_{k,MO} = 1$) can be obtained from Supplementary Equation (10),

$$E_{\theta,MO}^{\pm} = \frac{-i\Omega^2 g}{k^2 - (\alpha_{MO}^{\pm})^2 - \Omega^2\epsilon_d}, \tag{14.1}$$

$$E_{z,MO}^{\pm} = \frac{ik\alpha_{MO}^{\pm}}{k^2 - \Omega^2\epsilon_d}. \tag{14.2}$$

2. Metal layer ($z < 0$)

In the metal, $\mathbb{M}_m\boldsymbol{E}_m = 0$ corresponds to,

$$\begin{bmatrix} \epsilon_m - \Omega^{-2}k_{z,m}^2 & 0 & \Omega^{-2}kk_{z,m} \\ 0 & \epsilon_m - \Omega^{-2}(k^2 + k_{z,m}^2) & 0 \\ kk_{z,m}\Omega^{-2} & 0 & \epsilon_m - \Omega^{-2}k^2 \end{bmatrix} \begin{bmatrix} 1 \\ E_{\theta,m} \\ E_{z,m} \end{bmatrix} = \begin{bmatrix} 0 \\ 0 \\ 0 \end{bmatrix}, \tag{15}$$

where again we have chosen $E_{k,m} = 1$. This leads to the secular equation which yields the exponentially decaying field for $z < 0$; by letting $k_{z,m} = -i\alpha_m$,

$$\alpha_m = \sqrt{k^2 - \Omega^2\epsilon_m}. \tag{16}$$

Supplementary Equation (15) reveals that

$$E_{z,m} = -\frac{ik}{\alpha_m} \tag{17}$$

but does not inform us about the tangential component $E_{\theta,m}$ as the entry $(\mathbb{M}_m)_{\theta\theta} = \epsilon_m - \Omega^{-2}(k^2 + k^2_{z,m}) = 0$. This missing component will be deduced by matching the electromagnetic fields at the boundary $z = 0$.

D. Matching the modes at the boundary ($z = 0$)

We are looking for magneto-SP modes labeled by a propagating wavevector k and frequency $\omega(\boldsymbol{k})$, but, in general, different k_z evanescent wavevectors for each layer, which we have denoted $k^{\pm}_{z,MO} = i\alpha^{\pm}_{MO}$ and $k_{z,m} = -i\alpha_m$. The energy $\omega(\boldsymbol{k})$ (and therefore Ω) is unknown.

To summarize, for each pair (k,ω), there are two possible modes in the MO layer associated with different decay constants $\alpha^{\pm}_{MO}$ (see Supplementary Equation (13), $z > 0$),

$$\begin{aligned}
\vec{E}^{\pm}_{MO} &= \boldsymbol{E}^{\pm}_{MO}\eta e^{-\alpha^{\pm}_{MO}z} \\
&= (1, E^{\pm}_{\theta,MO}, E^{\pm}_{z,MO})\eta e^{-\alpha^{\pm}_{MO}z}, && (18.1)\\
\vec{B}^{\pm}_{MO} &= \boldsymbol{B}^{\pm}_{MO}\eta e^{-\alpha^{\pm}_{MO}z} \\
&= \frac{-i}{\omega}(\alpha^{\pm}E^{\pm}_{\theta,MO}, -ikE^{\pm}_{z,MO} - \alpha^{\pm}_{MO}, ikE^{\pm}_{\theta,MO})\eta e^{-\alpha^{\pm}_{MO}z}, && (18.2)
\end{aligned}$$

where the magnetic induction in Supplementary Equation (18.2) has been deduced from Supplementary Equation (18.1) and Maxwell's Supplementary Equation (3). The vectors have been written in cylindrical coordinates and we have defined $\eta \equiv e^{ikr-i\omega t}$. Hence, the total fields in the MO layer read,

$$\vec{E}_{MO} = t^{+}_{MO}\vec{E}^{+}_{MO} + t^{-}_{MO}\vec{E}^{-}_{MO}, \tag{19.1}$$
$$\vec{B}_{MO} = t^{+}_{MO}\vec{B}^{+}_{MO} + t^{-}_{MO}\vec{B}^{-}_{MO}. \tag{19.2}$$

where $t^{\pm}$ are coefficients to be determined. Similarly, for the metal layer ($z < 0$),

$$\begin{aligned}
\vec{E}_m &= \boldsymbol{E}_m\eta e^{\alpha_m z} \\
&= (1, E_{\theta,m}, E_{z,m})\eta e^{\alpha_m z}, && (20.1)\\
\vec{B}_m &= \boldsymbol{B}_m\eta e^{\alpha_m z} \\
&= \frac{-i}{\omega}(-\alpha_m E_{\theta,m}, -ikE_{z,m} + \alpha_m, ikE_{\theta,m})\eta e^{\alpha_m z}. && (20.2)
\end{aligned}$$

Here, we keep the arbitrary normalization where $E_{k,m} = 1$. Later on, we shall fix this normalization via quantization of the energy of the modes (see section F of this Supplementary Note). Furthermore, it is also safe to arbitrarily assume $E^{\pm}_{k,MO} = 1$ because Supplementary Equations (19.1) and (19.2) contain scaling coefficients $t^{\pm}$ which will be fixed by the boundary conditions at the metal-MO interface.

We are ready to match the fields at the interface at $z = 0$. The in-plane electric field and the perpendicular electric displacement each need to be continuous across the boundary: $E_{i,MO} = E_{i,m}$ for $i = r, \theta$, while $\epsilon_d E_{z,MO} = \epsilon_m E_{z,m}$. Furthermore, the magnetic field, and because $\overleftrightarrow{\mu} = 1$, its induction, are all continuous throughout, $B_{i,MO} = B_{i,m}$. These constraints altogether read,

$$1 = t^{+}_{MO} + t^{-}_{MO}, \tag{21.1}$$
$$E_{\theta,m} = t^{+}_{MO}E^{+}_{\theta,MO} + t^{-}_{MO}E^{-}_{\theta,MO}, \tag{21.2}$$
$$\epsilon_m E_{z,m} = \epsilon_d(t^{+}_{MO}E^{+}_{z,MO} + t^{-}_{MO}E^{-}_{z,MO}), \tag{21.3}$$
$$\alpha_m E_{\theta,m} = -t^{+}_{MO}\alpha^{+}_{MO}E^{+}_{\theta,MO} - t^{-}_{MO}\alpha^{-}_{MO}E^{-}_{\theta,MO}, \tag{21.4}$$
$$kE_{z,m} + i\alpha_m = t^{+}_{MO}(kE^{+}_{z,MO} - i\alpha^{+}_{MO}) + t^{-}_{MO}(kE^{-}_{z,MO} - i\alpha^{-}_{MO}), \tag{21.5}$$
$$E_{\theta,m} = t^{+}_{MO}E^{+}_{\theta,MO} + t^{-}_{MO}E^{-}_{\theta,MO}. \tag{21.6}$$

These constraints read similarly to the ones derived by Chiu and Quinn in [13] (see their Supplementary Equations (39)–(44)), except for the different coordinate conventions. Clearly, Supplementary Equations (21.2) and (21.6) are identical. Furthermore, Supplementary Equations (21.3) and (21.5) contain the same information, as can be shown by using Supplementary Equations (16) (14.2), (17), and (21.1). The remaining constraints yield the equation,

$$k^2 - \Omega^2\epsilon_d + \alpha^+_{MO}\alpha^-_{MO} + (\alpha^+_{MO} + \alpha^-_{MO})\alpha_m](k^2 - \Omega^2\epsilon_d)\epsilon_m \\ +\alpha_m\epsilon_d\{\alpha^+_{MO}\alpha^-_{MO}(\alpha^+_{MO} + \alpha^-_{MO}) + \alpha_m[(\alpha^+_{MO})^2 + \alpha^+_{MO}\alpha^-_{MO} + (\alpha^-_{MO})^2] - \alpha_m(k^2 - \Omega^2\epsilon_d)\} = 0. \tag{22}$$

By inserting Supplementary Equations (13), (16) into Supplementary Equation (22), we obtain a nonlinear equation in Ω for every value of $\boldsymbol{k}$. This equation can be numerically solved, at least in principle. Ω can be then used as input to Supplementary Equations (14.1), (14.2), (17), (19.1), (19.2), and (21.1)–(21.6) to solve for the electromagnetic modes. As we shall see, the most important qualitative feature of the solution of this problem is that the magneto-SP fields acquire tangential components (see Supplementary Equations (14.1) and (21.2)) which are absent when $g = 0$ [14, 15], that is, in the absence of an external magnetic field. Supplementary Equation (22) is identical to Supplementary Equation (45) in [13] upon carrying out the substitutions $\epsilon_{xx} = \epsilon_{zz} = \epsilon_d$.

E. Perturbation expansion on g

Chiu and Quinn reported a dispersion relation Ω *vs* $\boldsymbol{k}$ by numerically solving Supplementary Equation (22) for a very similar setup to the one of our interest. However, a detailed description of the resulting electromagnetic modes was not presented in that work. As explained, one may in principle solve for the profile of the electromagnetic modes once this dispersion is known. However, a numerical attempt at the problem using a standard nonlinear solver yielded spurious results for the modes.

Since the solution to the SP problem with no magnetic field ($g = 0$) is a well-known textbook result, and g is anyway much smaller than ϵ_d in a realistic setup, we may use a perturbation expansion of the equation in powers of g. Our goal is to obtain the electric fields up to $O(g)$, so that we can compute the magnitude of the exciton-SP coupling to that same order. To accomplish such objective, we first need to solve for Ω as well as the coefficients $t^{\pm}_{MO}$ up to $O(g)$. As we shall see, however, knowledge of $t^{\pm}_{MO}$ requires information about Ω up to $O(g^2)$. Once this is done, we simply Taylor expand the fields (18.1), 918.2), (20.1), (20.2) and collect the results according to Supplementary Equations (19.1)–(20.2)). In retrospect, the original problem we faced by trying to directly solve Supplementary Equation (22) originated from the fact that we were using the very small $O(g^2)$ corrections to Ω as an input to solve for the $O(g)$ electromagnetic modes. This requires an accurate solution of Ω, which is complicated by the highly nonlinear dependence of Supplementary Equation (22) on Ω. As a future consideration, it might be worth exploring numerical methodologies to attack this problem beyond the perturbative regime, although for our purposes, the latter suffices.

Even though the algebra below seems involved, it is straightforward to derive using a symbolic algebra package such as Wolfram Mathematica(c).

1. *Solving for* Ω

Given that the right hand side of Supplementary Equation (22) is zero, the polynomials at each power of g must each vanishing identically.

To start with and as a consistency check, at zeroth order in g, Supplementary Equation (22) becomes

$$[2\alpha^2_{d0} + 2\alpha_{d0}\alpha_{m0}]\alpha^2_{d0}\epsilon_{m0} + \alpha_{m0}\epsilon_{d0}\{\alpha^2_{d0}(2\alpha_{d0}) + \alpha_{m0}[3\alpha^2_{d0}] - \alpha_m\alpha^2_{d0}\} = \\ [2\alpha^3_{d0} + 2\alpha^2_{d0}\alpha_{m0}](\alpha_{d0}\epsilon_{m0} + \alpha_{m0}\epsilon_{d0}) = 0, \tag{23}$$

where the 0-subscripted variables denote the corresponding functions in Supplementary Equation (6), (13), (16) taking $\Omega = \Omega_0$,

$$\epsilon_{m0} = \epsilon_\infty - \frac{\Omega_P^2}{\Omega_0^2}, \tag{24.1}$$

$$\alpha_{d0} = \sqrt{k^2 - \epsilon_d\Omega_0^2}, \tag{24.2}$$

$$\alpha_{m0} = \sqrt{k^2 - \epsilon_{m0}\Omega_0^2}, \tag{24.3}$$

where $\Omega_P \equiv \frac{\omega_P}{c}$. Supplementary Equation (23) implies that either

$$2\alpha_{d0}^3 + 2\alpha_{d0}^2\alpha_{m0} = 0, \tag{25}$$

or

$$\alpha_{d0}\epsilon_{m0} + \alpha_{m0}\epsilon_d = 0. \tag{26}$$

The condition in Supplementary Equation (25) requires that $\alpha_{d0} = -\alpha_{m0}$, contradicting the very nature of the SP solution we are looking for, where $\alpha_{d0}, \alpha_{m0} > 0$ represent evanescent fields. However, the condition in Supplementary Equation (26) is simply the standard equation for the dispersion relation of a SP at the interface of an unmagnetized MO sample and a metal film [14, 15]. It can be readily solved yielding,

$$k = \Omega_0\sqrt{\frac{\epsilon_{m0}\epsilon_d}{\epsilon_{m0}+\epsilon_d}}, \tag{27}$$

or more explicitly,

$$\Omega_0 = \sqrt{\frac{\epsilon_d\Omega_P^2 + k^2(\epsilon_d+\epsilon_\infty) - \sqrt{[\epsilon_d\Omega_P^2 + k^2(\epsilon_d+\epsilon_\infty)]^2 - 4\epsilon_\infty\epsilon_d k^2\Omega_P^2}}{2\epsilon_\infty\epsilon_d}}. \tag{28}$$

At short k, the (linear) dispersion is very light-like, $\Omega_0 = \frac{k}{\sqrt{\epsilon_d}}$, and at large k, it plateaus to $\Omega_0 \to \frac{\Omega_P}{\sqrt{\epsilon_d+\epsilon_\infty}}$, corresponding to collective charge oscillations in the metal (see Supplementary Figure 2).

Moving on to the g^1 terms of Supplementary Equation (22) yields an equation of the form $\Omega^{(1)} f = 0$ where $f(\alpha_{d0}, \alpha_{m0})$ is a nonzero polynomial in α_{d0} and α_{m0}, implying that $\Omega^{(1)} = 0$. This result can be quickly derived as follows: the g^1 terms stem only from the power expansion of α_{MO}^+, α_{MO}^-, α_m, and ϵ_m. Some g^1 contributions from α_{MO}^+, α_{MO}^- are proportional to Ω_1 but some are not. Regardless, the ones from α_{MO}^+ come with the opposite sign to the ones from α_{MO}^-; hence, they vanish identically as Supplementary Equation (22) is symmetric in α_{MO}^+ and α_{MO}^-. On the other hand, every g^1 term for α_m and ϵ_m is strictly proportional to Ω_1 requiring $\Omega_1 = 0$ for the all the g^1 terms to cancel. Hence, the lowest order correction to Ω_0 of Ω arises at $O(g^2)$, that is,

$$\Omega \approx \Omega_0 + g^2\Omega_2. \tag{29}$$

As mentioned, we are solely interested in the calculation of the electric fields in each layer up to $O(g)$. However, as we shall see in the next section of this Supplementary Note, these corrections depend on Ω_2. To obtain this coefficient, we expand α_{MO}^+, α_{MO}^-, α_m, and ϵ_m up to $O(g^2)$, but not beyond that,

$$\alpha_{MO}^{\pm} \approx \alpha_{d0} \pm g\alpha_{d1} + g^2(\alpha_{d20} + \alpha_{d22}\Omega_2), \tag{30.1}$$

$$\alpha_m \approx \alpha_{m0} + g^2\alpha_{m22}\Omega_2, \tag{30.2}$$

$$\epsilon_m \approx \epsilon_{m0} + g^2\epsilon_{m22}\Omega_2, \tag{30.3}$$

where the coefficients in the expansions take the form,

$$\alpha_{d1} = \frac{i\Omega_0}{2\sqrt{\epsilon_d}}, \tag{31.1}$$

$$\alpha_{d20} = \frac{\Omega_0^2}{8\epsilon_d\alpha_{d0}}, \tag{31.2}$$

$$\alpha_{d22} = \frac{\Omega_0\epsilon_d}{\alpha_{d0}}, \tag{31.3}$$

$$\alpha_{m22} = -\frac{\Omega_0\epsilon_i}{\alpha_{m0}}, \tag{31.4}$$

$$\epsilon_{m22} = \frac{2\Omega_P^2}{\Omega_0^3}. \tag{31.5}$$

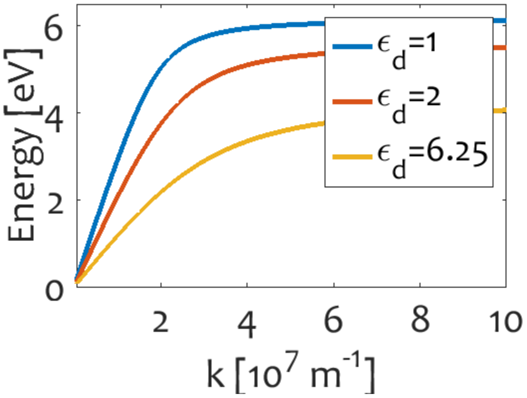

Supplementary Figure 2. *SP dispersion energy as a function of $k = |\boldsymbol{k}|$ for the two-layer (metal-MO dielectric) setup, assuming $g = 0$.* Plots generated using Supplementary Equation (28) with Drude parameters for Ag, $\epsilon_\infty \sim 4$, $\omega_P \sim 9$ eV, and varying the dielectric permittivity ϵ_d. Since $\Omega = \Omega_0 + O(g^2)$, the plots are correct up to $O(g)$, which is our perturbation order of interest. Notice that the dispersion curves start-off linearly (light-like excitations), but plateau to constant values at large wavevectors (charge oscillations in the metal), which become smaller as the dielectric permittivity increases.

Notice that in order to ultimately solve for Ω_2, we have separated the g^2 terms into two categories: those which are proportional to Ω_2 ($g^2\alpha_{d22}\Omega_2$, $g^2\alpha_{m22}\Omega_2$, $g^2\epsilon_{m22}\Omega_2$) and those that are not ($g^2\alpha_{d20}$). We substitute these expressions into Supplementary Equation (22) and collect the g^2 terms, which ought to cancel. The manipulations yield in a linear equation for Ω_2 which gives,

$$\Omega_2 = \frac{\Omega_0^5\epsilon_{m0}^2(3\epsilon_d - \epsilon_{m0})}{8\epsilon_d(\epsilon_d - \epsilon_{m0})\left[\Omega_P^2(2\alpha_{d0}^2\epsilon_{m0} + \Omega_0^2\epsilon_d^2) + \Omega_0^4\epsilon_d\epsilon_{m0}(\epsilon_d - \epsilon_{m0})\right]}, \tag{32}$$

where we have also used Supplementary Equation (26) in the form of $\alpha_{m0} = -\frac{\alpha_{d0}\epsilon_{m0}}{\epsilon_d}$ to simplify the final expression.

2. *Solving for $t^{\pm}_{MO}$*

Next, we expand the coefficient t^+_{MO}, which denotes the contribution of the + fields in Supplementary Equations (19.1) and (19.2) (t^-_{MO} can be subsequently found using the constraint in Supplementary Equation (21.1)),

$$t^+_{MO} \approx t_{d0} + g t_{d1}. \tag{33}$$

Here, t_{d0} and t_{d1} are unknown, but we shall solve for them using the boundary condition on the perpendicular electric fields (see Supplementary Equation (21.3)). We expand $E^{\pm}_{z,MO}$ and E_{zm} in Supplementary Equations (14.2) and (17) up to $O(g^2)$. Here, as opposed to Suplementary Equations (30.1)–(30.3), we do not separate the g^2 terms in $E^{\pm}_{z,MO}$ and E_{zm} (g^2E_{zd2} and g^2E_{zm2}) into those contributions which are proportional to Ω_2 and those which are not, as Ω_2 has already been determined in Supplementary Equation (32). The result is,

$$E^{\pm}_{z,MO} \approx E_{z,d0} \pm gE_{z,d1} + g^2E_{z,d2}, \tag{34.1}$$

$$E_{z,m} \approx E_{z,m0} + g^2E_{z,m2}, \tag{34.2}$$

where,

$$E_{z,d0} = \frac{ik}{\alpha_{d0}}, \tag{35.1}$$

$$E_{z,m0} = -\frac{ik}{\alpha_{m0}}, \tag{35.2}$$

$$E_{z,d1} = -\frac{k\Omega_0}{2\alpha_{d0}^2\sqrt{\epsilon_d}}, \tag{35.3}$$

$$E_{z,d2} = \frac{i(\alpha_{d0}\alpha_{d20}k + \alpha_{d0}\alpha_{d22}k\Omega_2 + 2k\Omega_0\Omega_2\epsilon_d)}{\alpha_{d0}^3}, \tag{35.4}$$

$$E_{z,m2} = -\frac{ik\Omega_0\Omega_2\epsilon_i}{\alpha_{m0}^3}. \tag{35.5}$$

We plug these expressions into Supplementary Equation (21.3). At zeroth-order in g, we get $\epsilon_{m0}E_{zm0} = \epsilon_d E_{zd0}$, which is equivalent to Supplementary Equation (26) and does not give us information on t_{d0} or t_{d1} (this indeterminacy ultimately reveals why we need expansions up to $O(g^2)$ to obtain fields up to $O(g)$). At $O(g)$ we get the intuitive result,

$$t_{d0} = \frac{1}{2}, \tag{36}$$

and at $O(g^2)$ we obtain,

$$\begin{aligned} t_{d1} &= \frac{-E_{z,d2}\epsilon_d + E_{z,m0}\Omega_2\epsilon_{m22} + E_{z,m2}\epsilon_{m0}}{2E_{zd1}\epsilon_d} \\ &= i\underbrace{\left(\frac{16\alpha_{d0}^3\alpha_{m0}^2\Omega_2\Omega_P^2 + 8\alpha_{d0}^3\Omega_0^4\Omega_2\epsilon_\infty\epsilon_{m0} + \alpha_{m0}^3\Omega_0^5 + 8\alpha_{m0}^3\Omega_0^4\Omega_2\epsilon_d^2}{8\alpha_{d0}\alpha_{m0}^3\Omega_0^4\sqrt{\epsilon_d}}\right)}_{\equiv\tau_{d1}}, \end{aligned} \tag{37}$$

which, together with Ω_2 in Supplementary Equation (32), can be readily evaluated with zeroth-order parameters. We notice that t_{d1} is purely imaginary-valued, so we have written it in terms of a purely real-valued τ_{d1}. Given Supplementary Equation (33), it is clear from Supplementary Equation (21.1) that

$$t_{MO}^- \approx t_{d0} - g t_{d1}. \tag{38}$$

3. *Collecting the expressions for the fields*

We now have all the ingredients to evaluate $\vec{E}_{MO}$ and $\vec{B}_{MO}$ (see Supplementary Equations (19.1)–(19.2)) up to $O(g)$,

$$\vec{E}_{MO} \approx \vec{E}_{d0} + g\vec{E}_{d1}, \tag{39.1}$$

$$\vec{B}_{MO} \approx \vec{B}_{d0} + g\vec{B}_{d1}, \tag{39.2}$$

where, at zeroth-order, in the absence of external magnetic field, we have the standard SP mode which is a transverse magnetic (TM) mode [14, 15],

$$\vec{E}_{d0} = \underbrace{\left(1, 0, \frac{ik}{\alpha_{d0}}\right)}_{\equiv \boldsymbol{E}_{d0}} \eta_0 e^{-\alpha_{d0}z}, \tag{40.1}$$

$$\begin{aligned} \vec{B}_{d0} &= \frac{-i}{\Omega_0 c}\left(0, \frac{k^2}{\alpha_{d0}} - \alpha_{d0}, 0\right)\eta_0 e^{-\alpha_{d0}z} \\ &= \underbrace{-\frac{i\epsilon_d\Omega_0}{\alpha_{d0}c}(0,1,0)}_{\equiv \boldsymbol{B}_{d0}}\eta_0 e^{-\alpha_{d0}z}. \end{aligned} \tag{40.2}$$

In going from the first to the second line of Supplementary Equation (40.2), we have used Supplementary Equation (24.2). Supplementary Equations (40.1)–(40.2) feature an elliptically-polarized electric field with no tangential component, and a purely tangential and imaginary-valued magnetic induction. The opposite is true for the first-order correction:

it consists of a purely tangential and imaginary-valued electric field (recall that t_{d1} is purely imaginary, see Supplementary Equation (37)) and an elliptically polarized electric field with no tangential part,

$$\vec{E}_{d1} = \underbrace{\frac{i\Omega_0(4\tau_{d1}\sqrt{\epsilon_d} - \Omega_0 z)}{2\alpha_{d0}}(0,1,0)}_{\equiv \boldsymbol{E}_{d1}} \eta_0 e^{-\alpha_{d0}z}, \tag{41.1}$$

$$\vec{B}_{d1} = \underbrace{\frac{1}{2\alpha_{d0}c}\Big(-4\alpha_{d0}\tau_{d1}\sqrt{\epsilon_d} + \Omega_0(1-\alpha_{d0}z), 0, ik(4\tau_{d1}\sqrt{\epsilon_d} - \Omega_0 z)\Big)}_{\equiv \boldsymbol{B}_{d1}} \eta_0 e^{-\alpha_{d0}z}. \tag{41.2}$$

In deriving Supplementary Equations (39.1)–(41.1), we have used the $O(g)$ Taylor expansion for the fields (see Supplementary Equations (14.1)–(14.2), (18.2)). Notice that besides the exponentially decreasing dependence of the fields, we also obtain a polynomial contribution in z. We already computed some of the relevant expansion coefficients in Supplementary Equations (34.1)–(35.3); the remaining ones that we used are,

$$E^{\pm}_{\theta,MO} \approx \pm\frac{\Omega_0\sqrt{\epsilon_d}}{\alpha_{d0}}, \tag{42.1}$$

$$B^{\pm}_{r,MO} \approx \mp\frac{i\sqrt{\epsilon_d}}{c} + g\frac{\Omega_0}{2\alpha_{d0}c}, \tag{42.2}$$

$$B^{\pm}_{\theta,MO} \approx -\frac{i\Omega_0\epsilon_d}{\alpha_{d0}c} + g\frac{\Omega_0^2\sqrt{\epsilon_d}}{2\alpha_{d0}^2 c}, \tag{42.3}$$

$$B^{\pm}_{z,MO} \approx \pm\frac{k\sqrt{\epsilon_d}}{\alpha_{d0}c}, \tag{42.4}$$

as well as those for the plane wave components,

$$\begin{aligned}\eta e^{-\alpha^{\pm}_{MO}z} &= e^{i(kr-\omega t)-\alpha^{\pm}_{MO}z} \\ &\approx \underbrace{e^{i(kr-c\Omega_0 t)}}_{\equiv \eta_0} e^{-\alpha_{d0}z}\Big(1 \mp g\frac{i\Omega_0}{2\sqrt{\epsilon_d}}z\Big).\end{aligned} \tag{43}$$

Finally, given $\vec{E}_{MO}$, $\vec{E}_m$ can be readily obtained from the boundary conditions for the fields at $z = 0$ (see Supplementary Equations (21.1)–(21.6)) as well as the original ansatz for their functional forms (see Supplementary Equation (20.1)–(20.2)),

$$\vec{E}_m \approx \vec{E}_{m0} + g\vec{E}_{m1}, \tag{44}$$

where, at zeroth-order we have the standard SP electric field as if there were no magnetic field present,

$$\vec{E}_{m0} = \Big(1, 0, \frac{\epsilon_d}{\epsilon_{m0}}\frac{ik}{\alpha_{d0}}\Big)\eta_0 e^{\alpha_{m0}z} = \underbrace{\Big(1, 0, -\frac{ik}{\alpha_{m0}}\Big)}_{\equiv \boldsymbol{E}_{m0}}\eta_0 e^{\alpha_{m0}z}, \tag{45.1}$$

$$\vec{B}_{m0} = \frac{i\epsilon_{m0}\Omega_0}{\alpha_{m0}c}(0,1,0)\eta_0 e^{\alpha_{m0}z} = \underbrace{-\frac{i\epsilon_d\Omega_0}{\alpha_{d0}c}(0,1,0)}_{\equiv \boldsymbol{B}_{m0}}\eta_0 e^{\alpha_{m0}z}. \tag{45.2}$$

Here, we have used Supplementary Equation (26) in both lines. Similarly, the first order correction to $\vec{E}_{m0}$ is,

$$\vec{E}_{m1} = \underbrace{\frac{2i\tau_{d1}\Omega_0\sqrt{\epsilon_d}}{\alpha_{d0}}(0,1,0)}_{\equiv \boldsymbol{E}_{m1}}\eta_0 e^{\alpha_{m0}z}, \tag{46}$$

$$\vec{B}_{m1} = \underbrace{\frac{2\tau_{d1}}{\alpha_{d0}c}\Big(\Omega_0 - \alpha_{d0}\sqrt{\epsilon_d}, 0, ik\sqrt{\epsilon_d}\Big)}_{\equiv \boldsymbol{B}_{m1}}\eta_0 e^{\alpha_{m0}z}.$$

F. Quantization and normalization of modes

The previous section of this Supplementary Note shows how to (perturbatively) compute the frequency Ω, the electric field, and the magnetic induction of an SP mode with wavevector $\boldsymbol{k}$. Note that, so far, we have invoked an arbitrary normalization (setting $E_r = 1$). To fix this, we first ought to compute the energy associated with the unnormalized modes. Consider placing electric field amplitude $\mathscr{A}_{\boldsymbol{k}}$ into the radial component of the $\boldsymbol{k}$ mode. The energy in this mode is quadratic in the fields (note that even if the electric field is much larger than the magnetic induction, the prefactors of ϵ_0 and $\frac{1}{\mu_0}$ weigh them in a way that their contributions to the energy density are on the same order of magnitude) [15, 16],

$$H_{SP,\boldsymbol{k}} = \frac{1}{2}\sum_i \int dV \left[\epsilon_0 \sum_j \frac{d(\omega\epsilon^*_{ij}(\omega))}{d\omega}(\vec{E})^*_j(\vec{E})_i + \frac{1}{\mu_0\mu}|(\vec{B})_i|^2\right]|\mathscr{A}_{\boldsymbol{k}}|^2, \tag{47}$$

where $i, j \in \{r, \theta, z\}$, and electric field and magnetic inductions in each $\boldsymbol{k}$ mode (throughout z) are conveniently written as,

$$\vec{E} = \Theta(-z)\vec{E}_m + \Theta(z)\vec{E}_{MO}, \tag{48.1}$$
$$\vec{B} = \Theta(-z)\vec{B}_m + \Theta(z)\vec{B}_{MO}, \tag{48.2}$$

and $(\vec{E})_i$, $(\vec{B})_i$ denote the i-th components of the respective fields (which include the plane wave exponential factors, see (19.1)–(20.2)). In Supplementary Equation (47), we have absorbed the electric field units into $\mathscr{A}_{\boldsymbol{k}}$, so E_i is taken to be a unitless quantity. The integration $\int dV$ is carried out over all 3D space.

Assuming a finite size box of in-plane area S and infinite perpendicular dimension and plugging in Supplementary Equations (19.1), (19.2), (20.1), and (20.2) into Supplementary Equation (47), we obtain,

$$\begin{aligned}
H_{SP,\boldsymbol{k}} &= \frac{S|\mathscr{A}_{\boldsymbol{k}}|^2}{2}\sum_i \int_{-\infty}^{\infty} dz\left(\epsilon_0 \sum_j \frac{d(\omega\epsilon^*_{ij}(\omega))}{d\omega}(\vec{E})^*_j(\vec{E})_i + \frac{1}{\mu_0\mu}|(\vec{B})_i|^2\right) \\
&= \frac{S|\mathscr{A}_{\boldsymbol{k}}|^2}{2}\sum_i \sum_{\gamma,\delta\in\{+,-\}} (t^\gamma_{MO})^* t^\delta_{MO}\left[\epsilon_0\sum_j \epsilon^*_{MO,ij}(E^\gamma_{j,MO})^* E^\delta_{i,MO} + \frac{1}{\mu_0\mu}(B^\gamma_{i,MO})^* B^\delta_{i,MO}\right] \\
&\quad \times \left[\int_0^\infty dz e^{-[(\alpha^\gamma_{MO})^* + \alpha^\delta_{MO}]z}\right] \\
&\quad + \frac{S|\mathscr{A}_{\boldsymbol{k}}|^2}{2}\sum_i \left[\epsilon_0 \frac{d(\omega\epsilon_m(\omega))}{d\omega}|E_{i,m}|^2 + \frac{1}{\mu_0\mu}|B_{i,m}|^2\right]\left[\int_{-\infty}^0 dz e^{(\alpha^*_0+\alpha_0)z}\right] \\
&= S|\mathscr{A}_{\boldsymbol{k}}|^2 \sum_i \sum_{\gamma,\delta\in\{+,-\}} (t^\gamma_{MO})^* t^\delta_{MO}\left[\epsilon_0\sum_j \epsilon^*_{MO,ij}(E^\gamma_{j,MO})^* E^\delta_{i,MO} + \frac{1}{\mu_0\mu}(B^\gamma_{i,MO})^* B^\delta_{i,MO}\right]\frac{1}{2(\alpha^*_\gamma + \alpha_\delta)} \\
&\quad + S|\mathscr{A}_{\boldsymbol{k}}|^2 \sum_i \left[\epsilon_0 \frac{d(\omega\epsilon_m(\omega))}{d\omega}|E_{i,m}|^2 + \frac{1}{\mu_0\mu}|B_{i,m}|^2\right]\frac{1}{2(\alpha^*_m + \alpha_m)} \\
&\equiv S\left(\frac{\epsilon_0 L_{\boldsymbol{k}}}{4}\right)\left(2|\mathscr{A}_{\boldsymbol{k}}|^2\right) \\
&= S\frac{\epsilon_0 L_{\boldsymbol{k}}}{4}(\mathscr{A}_{\boldsymbol{k}}\mathscr{A}^*_{\boldsymbol{k}} + \mathscr{A}^*_{\boldsymbol{k}}\mathscr{A}_{\boldsymbol{k}}),
\end{aligned} \tag{49}$$

where, following [16], we have defined the vertical mode length as,

$$\begin{aligned}
L_{\boldsymbol{k}} &= \sum_i \sum_{\gamma,\delta\in\{+,-\}} (t^\gamma_{MO})^* t^\delta_{MO}\left[\epsilon_0\sum_j \epsilon^*_{MO,ij}(E^\gamma_{j,MO})^* E^\delta_{i,MO} + \frac{1}{\mu_0\mu}(B^\gamma_{i,MO})^* B^\delta_{i,MO}\right]\frac{1}{\epsilon_0(\alpha^*_\gamma + \alpha_\delta)} \\
&\quad + \sum_i \left[\epsilon_0 \frac{d(\omega\epsilon_m(\omega))}{d\omega}|E_{i,m}|^2 + \frac{1}{\mu_0\mu}|B_{i,m}|^2\right]\frac{1}{\epsilon_0(\alpha^*_m + \alpha_m)}.
\end{aligned} \tag{50}$$

Since E_i is taken to be unitless, $L_{\boldsymbol{k}}$ effectively has units of length. Physically, $L_{\boldsymbol{k}}$ defines a mode volume $SL_{\boldsymbol{k}}$ with a quantized amount of energy corresponding to the frequency $\omega_{\boldsymbol{k}}$. Importantly Supplementary Equation (49) is quadratic in the electric field amplitude $\mathscr{A}_{\boldsymbol{k}}$, which implies that each $\boldsymbol{k}$ mode corresponds to a harmonic oscillator. If we wish to quantize the energy in quanta of $\omega_{\boldsymbol{k}}$,

$$H_{SP,\boldsymbol{k}} = \frac{\omega(\boldsymbol{k})}{2}(\alpha_{\boldsymbol{k}}\alpha^*_{\boldsymbol{k}} + \alpha^*_{\boldsymbol{k}}\alpha_{\boldsymbol{k}}), \tag{51}$$

we can define $\alpha_{\boldsymbol{k}}$ so that,

$$\mathscr{A}_{\boldsymbol{k}} = \sqrt{\frac{\omega(\boldsymbol{k})}{SL_{\boldsymbol{k}}}}\alpha_{\boldsymbol{k}}. \tag{52}$$

Other multiplicative phase factors in this amplitude definition (U(1) gauge choice) do not affect the quantization. Promoting the complex amplitudes to operators, $\alpha_{\boldsymbol{k}} \to a_{\boldsymbol{k}}$ and $\alpha^*_{\boldsymbol{k}} \to a^\dagger_{\boldsymbol{k}}$ with $[a_{\boldsymbol{k}}, a^\dagger_{\boldsymbol{k}}] = 1$,

$$\begin{aligned} H_{SP,\boldsymbol{k}} &= \frac{\omega(\boldsymbol{k})}{2}(a_{\boldsymbol{k}}a^\dagger_{\boldsymbol{k}} + a^\dagger_{\boldsymbol{k}}a_{\boldsymbol{k}}) \\ &= \omega(\boldsymbol{k})\Big(a_{\boldsymbol{k}}a^\dagger_{\boldsymbol{k}} + \frac{1}{2}\Big). \end{aligned} \tag{53}$$

To summarize, we have normalized each $\boldsymbol{k}$ mode in Supplementary Equations (48.1) and (48.2) by associating energy quanta $\omega_{\boldsymbol{k}}$ to a SP excitation in such mode. Note that in the main text, the Hamiltonian H_{SP} ignores the zero-point energy $\frac{\omega(\boldsymbol{k})}{2}$ for each mode since the energy gap of the excitons is also given relative to a ground state. Finally, the final electric field and magnetic induction are superpositions of amplitudes in such modes,

$$\vec{\mathscr{E}} = \sum_{\boldsymbol{k}} \mathscr{A}_{\boldsymbol{k}}\vec{E}(\boldsymbol{k}). \tag{54.1}$$

$$\vec{\mathscr{B}} = \sum_{\boldsymbol{k}} \mathscr{A}_{\boldsymbol{k}}\vec{B}(\boldsymbol{k}). \tag{54.2}$$

Promoting these amplitudes to operators (in the Heisenberg picture) and using Supplementary Equation (52),

$$\begin{aligned} \hat{\vec{\mathscr{E}}}(\boldsymbol{r},t) &= \sum_{\boldsymbol{k}} 2\sqrt{\frac{\omega(\boldsymbol{k})}{2\epsilon_0 SL_{\boldsymbol{k}}}}a_{\boldsymbol{k}}\vec{E}(\boldsymbol{k}) \\ &= \sum_{\boldsymbol{k}} \sqrt{\frac{\omega(\boldsymbol{k})}{2\epsilon_0 SL_{\boldsymbol{k}}}}a_{\boldsymbol{k}}\vec{E}(\boldsymbol{k}) + \sqrt{\frac{\omega(\boldsymbol{k})}{2\epsilon_0 SL_{\boldsymbol{k}}}}a^\dagger_{\boldsymbol{k}}\vec{E}^*(\boldsymbol{k}), \end{aligned} \tag{55.1}$$

$$\hat{\vec{\mathscr{B}}}(\boldsymbol{r},t) = \sum_{\boldsymbol{k}} \sqrt{\frac{\omega(\boldsymbol{k})}{2\epsilon_0 SL_{\boldsymbol{k}}}}a_{\boldsymbol{k}}\vec{B}(\boldsymbol{k}) + \sqrt{\frac{\omega(\boldsymbol{k})}{2\epsilon_0 SL_{\boldsymbol{k}}}}a^\dagger_{\boldsymbol{k}}\vec{B}^*(\boldsymbol{k}), \tag{55.2}$$

where we have used the fact that $\vec{\mathscr{E}}$ and $\vec{\mathscr{B}}$ are real valued to write the operators in a more conventionally symmetric form.

G. Perturbation expansion of $L_{\boldsymbol{k}}$

The fields deduced in section E of this Supplementary Note are only correct up to $O(g)$, so it is important that we keep $L_{\boldsymbol{k}}$ up to that order too. Importantly, since $(\overleftrightarrow{\epsilon}_{MO})_{r\theta} = ig$ and $E_{\theta d} \propto g$, the lowest order contribution to $L_{\boldsymbol{k}}$ appears at $O(g^2)$. Hence, we do not need to take the anisotropic effects of ϵ_{MO} into account, and we can set $g = 0$ in Supplementary Equation (7), i.e. $\overleftrightarrow{\epsilon}_{MO} \approx \epsilon_d \mathbb{I}$, where $\mathbb{I}$ is the 3×3 identity matrix. Therefore, the calculation for $L_{\boldsymbol{k}} \approx L_{\boldsymbol{k}0}$ reduces to that of the standard SP mode in the absence of external magnetic field (α_{d0} and α_{m0} are purely real) [16],

$$\begin{aligned} L_{\boldsymbol{k}0} &= \sum_i \left\{ \left[\epsilon_0\epsilon_d|E_{i,d0}|^2 + \frac{1}{\mu_0\mu}|B_{i,d0}|^2\right]\frac{1}{2\epsilon_0\alpha_{d0}} + \left[\epsilon_0 \frac{d(\omega\epsilon_{m0}(\omega))}{d\omega}\bigg|_{\omega=\frac{\Omega_0}{c}}|E_{i,m0}|^2 + \frac{1}{\mu_0\mu}|B_{i,m0}|^2\right]\frac{1}{2\epsilon_0\alpha_{m0}} \right\} \\ &= \left[\epsilon_d\left(1+\frac{k^2}{\alpha_{d0}^2}\right) + \left(\frac{\epsilon_d\Omega_0}{\alpha_{d0}}\right)^2\right]\frac{1}{2\alpha_{d0}} + \left[\frac{d(\omega\epsilon_{m0}(\omega))}{d\omega}\bigg|_{\omega=\frac{\Omega_0}{c}}\left(1+\frac{k^2}{\alpha_{m0}^2}\right) + \left(\frac{\epsilon_d\Omega_0}{\alpha_{d0}}\right)^2\right]\frac{1}{2\alpha_{m0}} \\ &= \frac{-\epsilon_{m0}}{\alpha_{d0}} + \frac{1}{2\alpha_{m0}}\left[\frac{d(\omega\epsilon_{m0}(\omega))}{d\omega}\bigg|_{\omega=\frac{\Omega_0}{c}}\left(\frac{\epsilon_{m0}-\epsilon_d}{\epsilon_{m0}}\right) - \epsilon_{m0} - \epsilon_d\right]. \end{aligned} \tag{56}$$

In this derivation, we have used $\mu = 1$, $\epsilon_0 \mu_0 = c^{-2}$, Supplementary Equations (27), (35.1), and (35.2) as well as the expressions for (24.2), and (24.3). The final result in Supplementary Equation (56) is twice of what is reported in the Supplementary Material of [16]. We believe our derivation has the correct prefactors; however, the use of either result gives the same order of magnitude of the effects we are interested in.

Supplementary Note 2. MAGNETO-SPS IN A THREE-LAYER SETUP

We shall now adapt the results from Supplementary Note 1 to the situation where the MO layer has a finite height a, and an organic layer of isotropic dielectric ϵ_{org} is placed on top of it (see Supplementary Figure 3). As far as we are aware, the resulting expressions for the corresponding magneto-SPs have not appeared before in the literature.

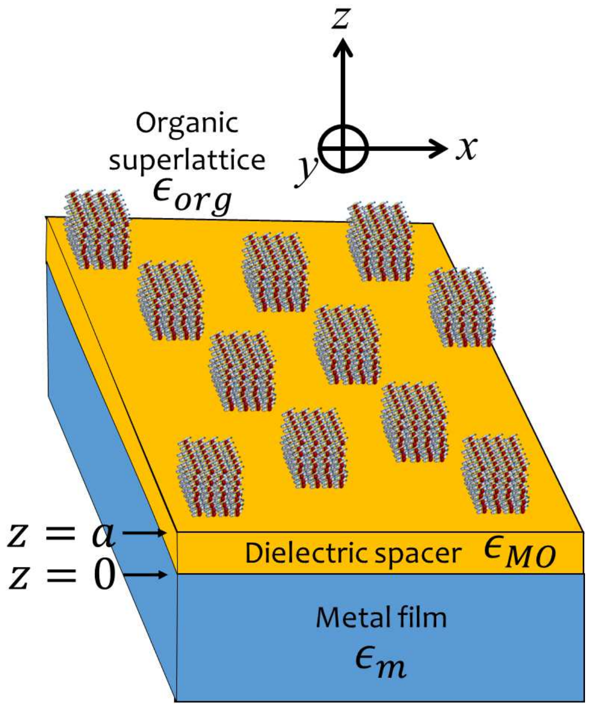

Supplementary Figure 3. *Three-layer (metal-MO dielectric-organic) setup.* We are interested in the (magneto)-SP modes arising at the dielectric-organic interface ($z = a$) upon application of a perpendicular external magnetic field.

A. Electromagnetic modes for each layer

1. Organic layer ($z > a$)

Just as we did for the MO and the metal layers (see Supplementary Equations (10) and (15)), Supplementary Equation (9) for the organic layer can be expressed in matrix form $\mathbb{M}_{org}\boldsymbol{E}_{org} = 0$,

$$\begin{bmatrix} \epsilon_m - \Omega^{-2}k_{z,org}^2 & 0 & \Omega^{-2}kk_{z,org} \\ 0 & \epsilon_m - \Omega^{-2}(k^2 + k_{z,org}^2) & 0 \\ kk_{z,org}\Omega^{-2} & 0 & \epsilon_m - \Omega^{-2}k^2 \end{bmatrix} \begin{bmatrix} E_{k,org} \\ E_{\theta,org} \\ E_{z,org} \end{bmatrix} = \begin{bmatrix} 0 \\ 0 \\ 0 \end{bmatrix}. \tag{57}$$

Importantly, we do not fix the radial component $E_{k,org}$ to 1 because of the boundary conditions at the organic crystal and MO interface at $z = a$. In our (arbitrary) normalization before quantization, we may set only one of the field components in one of the layers to 1, and our convention is to choose $E_{r,m} = 1$ as in the two-layer case. The rest of the fields are not arbitrary and satisfy the wave equation Supplementary Equation (9) as well as boundary conditions at each of the interfaces. The corresponding secular equation for the decaying field for $z > a$, with $k_{z,org} = i\alpha_{org}$ yields

$$\alpha_{org} = \sqrt{k^2 - \Omega^2\epsilon_{org}}, \tag{58}$$

as expected (see Supplementary Equation (16)). Finally, just like in the metal layer, Supplementary Equation (57) tells us that

$$E_{z,org} = \frac{ik}{\alpha_{org}} E_{r,org}, \tag{59}$$

but does not inform us about the tangential component $E_{\theta,org}$ (nor about $E_{k,org}$), The missing components will be deduced by matching the boundary at $z = a$. Altogether, the fields in the organic layer read like those in Supplementary Equations (18.1) and (18.2),

$$\begin{aligned}\vec{E}_{org} &= \boldsymbol{E}_{org}\eta e^{-\alpha_{org}z}\\ &= (E_{k,org}, E_{\theta,m}, E_{z,m})\eta e^{\alpha_m z}, \qquad (60.1)\\ \vec{B}_{org} &= \boldsymbol{B}_{org}\eta e^{-\alpha_{org}z}\\ &= \frac{-i}{\omega}(\alpha_{org}E_{\theta,org}, -ikE_{z,org} - \alpha_{org}E_{k,org}, ikE_{\theta,org})\eta e^{-\alpha_{org}z}, \qquad (60.2)\end{aligned}$$

denoting exponentially decreasing fields.

2. MO layer ($a > z > 0$)

For the MO layer, we translate Supplementary Equations (18.1) and (18.2) to this setup,

$$\begin{aligned}\vec{E}^{\pm}_{MO\downarrow} &= \boldsymbol{E}^{\pm}_{MO\downarrow}\eta e^{-\alpha^{\pm}z}\\ &= (1, E^{\pm}_{\theta,MO\downarrow}, E^{\pm}_{z,MO\downarrow})\eta e^{-\alpha^{\pm}z}, \qquad (61.1)\\ \vec{B}^{\pm}_{MO\downarrow} &= \boldsymbol{B}^{\pm}_{MO\downarrow}\eta e^{-\alpha^{\pm}z}\\ &\frac{-i}{\omega}(\alpha^{\pm}E^{\pm}_{\theta,MO\downarrow}, -ikE^{\pm}_{z,MO\downarrow} - \alpha^{\pm}, ikE^{\pm}_{\theta,MO\downarrow})\eta e^{-\alpha^{\pm}z}, \qquad (61.2)\end{aligned}$$

where $\vec{E}^{\pm}_{MO\downarrow}$ and $\vec{B}^{\pm}_{MO\downarrow}$ indicate exponentially decreasing fields ($k_z = i\alpha^{\pm}_{MO}$), and by slightly adapting these expressions,

$$\begin{aligned}\vec{E}^{\pm}_{MO\uparrow} &= \boldsymbol{E}^{\pm}_{MO\uparrow}\eta e^{\alpha^{\pm}z}\\ &= (1, E^{\pm}_{\theta,MO\uparrow}, E^{\pm}_{z,MO\uparrow})\eta e^{\alpha^{\pm}z}, \qquad (62.1)\\ \vec{B}^{\pm}_{MO\uparrow} &= \boldsymbol{B}^{\pm}_{MO\uparrow}\eta e^{\alpha^{\pm}z}\\ &\frac{-i}{\omega}(-\alpha^{\pm}E^{\pm}_{\theta,MO\uparrow}, -ikE^{\pm}_{z,MO\uparrow} + \alpha^{\pm}, ikE^{\pm}_{\theta,MO\uparrow})\eta e^{\alpha^{\pm}z}, \qquad (62.2)\end{aligned}$$

where $\vec{E}^{\pm}_{MO\uparrow}$ and $\vec{B}^{\pm}_{MO\uparrow}$ denote exponentially increasing fields ($k_z = -i\alpha^{\pm}_{MO}$). In Supplementary Note 1, where MO was considered to fill up all the space $z > 0$, the latter fields were not considered, the reason being that $e^{\alpha^{\pm}_{MO}z}$ was unbounded as $z \to \infty$; this is not the case when the largest value of z is a. A more intuitive way to describe this situation is that $\vec{E}^{\pm}_{BIG\downarrow}$ and $\vec{B}^{\pm}_{BIG\downarrow}$ denote fields that exponentially decrease starting from $z = 0$ going upwards; similarly, $\vec{E}^{\pm}_{BIG\uparrow}$ and $\vec{B}^{\pm}_{BIG\uparrow}$ describe exponentially decreasing fields starting from $z = a$ going downwards. Hence, the analogous expressions to Supplementary Equations (19.1) and (19.2) are,

$$\vec{E}_{MO} = t^{+}_{\downarrow}\vec{E}^{+}_{MO\downarrow} + t^{-}_{\downarrow}\vec{E}^{-}_{MO\downarrow} + t^{+}_{\uparrow}\vec{E}^{+}_{MO\uparrow} + t^{-}_{\uparrow}\vec{E}^{-}_{MO\uparrow}, \tag{63.1}$$

$$\vec{B}_{MO} = t^{+}_{\downarrow}\vec{B}^{+}_{MO\downarrow} + t^{-}_{\downarrow}\vec{B}^{-}_{MO\downarrow} + t^{+}_{\uparrow}\vec{B}^{+}_{MO\uparrow} + t^{-}_{\uparrow}\vec{B}^{-}_{MO\uparrow}. \tag{63.2}$$

where $t^{\pm}_{\downarrow}$ and $t^{\pm}_{\uparrow}$ are the unknown coefficients. Here, $E^{\pm}_{\theta,MO\downarrow} = E^{\pm}_{\theta,MO\uparrow} = E^{\pm}_{\theta,MO}$ but $E^{\pm}_{z,MO\downarrow} = -E^{\pm}_{z,MO\uparrow} = E^{\pm}_{z,MO}$. These identities are easy to check as $\vec{E}^{\pm}_{MO\downarrow}$ is associated with $k_z = i\alpha^{\pm}_{MO}$ and $\vec{E}^{\pm}_{MO\uparrow}$ with $k_z = -i\alpha^{\pm}_{MO}$, but Supplementary Equations (14.1) and (14.2) were derived for $k_z = i\alpha^{\pm}_{MO}$.

3. Metal layer ($z < 0$)

Finally, for the metal layer, all the expressions we derived in Sec. Supplementary Note 1 hold, in particular Supplementary Equations (20.1) and (20.2); we still assume $E_{k,m} = 1$.

B. Matching the modes at the boundaries ($z = 0$ and $z = a$)

Given this prelude, the analogous boundary conditions to Supplementary Equations (21.1)–(21.6) for $z = 0$ are,

$$1 = t^+_{MO\downarrow} + t^-_{MO\downarrow} + t^+_{MO\uparrow} + t^-_{MO\uparrow}, \tag{64.1}$$

$$E_{\theta,m} = t^+_{MO\downarrow}E^+_{\theta,MO\downarrow} + t^-_{MO\downarrow}E^-_{\theta,MO\downarrow} + t^+_{MO\uparrow}E^+_{\theta,MO\uparrow} + t^-_{MO\uparrow}E^-_{\theta,MO\uparrow}, \tag{64.2}$$

$$\epsilon_m E_{z,m} = \epsilon_d(t^+_{MO\downarrow}E^+_{z,MO\downarrow} + t^-_{MO\downarrow}E^-_{z,MO\downarrow} + t^+_{MO\uparrow}E^+_{z,MO\uparrow} + t^-_{MO\uparrow}E^-_{z,MO\uparrow}), \tag{64.3}$$

$$\alpha_m E_{\theta,m} = -(\alpha^+_{MO}t^+_{MO\downarrow}E^+_{\theta,MO\downarrow} + \alpha^-_{MO}t^-_{MO\downarrow}E^-_{\theta,MO\downarrow}) + (\alpha^+_{MO}t^+_{MO\uparrow}E^+_{\theta,MO\uparrow} + \alpha^-_{MO}t^-_{MO\uparrow}E^-_{\theta,MO\uparrow}), \tag{64.4}$$

$$\begin{aligned} kE_{z,m} + i\alpha_m = {} & k(t^+_{MO\downarrow}E^+_{z,MO\downarrow} + t^-_{MO\downarrow}E^-_{z,MO\downarrow} + t^+_{MO\uparrow}E^+_{zMO\uparrow} + t^-_{MO\uparrow}E^-_{z,MO\uparrow}) \\ & -i\alpha^+_{MO}(t^+_{MO\downarrow} - t^+_{MO\uparrow}) - i\alpha^-_{MO}(t^-_{MO\downarrow} - t^-_{MO\uparrow}), \end{aligned} \tag{64.5}$$

$$E_{\theta,m} = t^+_{MO\downarrow}E^+_{\theta,MO\downarrow} + t^-_{MO\downarrow}E^-_{\theta,MO\downarrow} + t^+_{MO\uparrow}E^+_{\theta,MO\uparrow} + t^-_{MO\uparrow}E^-_{\theta,MO\uparrow}, \tag{64.6}$$

whereas for $z = a$ they are,

$$E_{k,org} = t^+_{MO\downarrow}\chi^+ + t^-_{MO\downarrow}\chi^- + t^+_{MO\uparrow}\frac{1}{\chi^+} + t^-_{MO\uparrow}\frac{1}{\chi^-}, \tag{65.1}$$

$$E_{\theta,org} = t^+_{MO\downarrow}E^+_{\theta,MO\downarrow}\chi^+ + t^-_{MO\downarrow}E^-_{\theta,MO\downarrow}\chi^- + t^+_{MO\uparrow}E^+_{\theta,MO\uparrow}\frac{1}{\chi^+} + t^-_{MO\uparrow}E^-_{\theta,MO\uparrow}\frac{1}{\chi^-}, \tag{65.2}$$

$$\epsilon_{org}E_{z,org} = \epsilon_d\left(t^+_{MO\downarrow}E^+_{z,MO\downarrow}\chi^+ + t^-_{MO\downarrow}E^-_{z,MO\downarrow}\chi^- + t^+_{MO\uparrow}E^+_{z,MO\uparrow}\frac{1}{\chi^+} + t^-_{MO\uparrow}E^-_{z,MO\uparrow}\frac{1}{\chi^-}\right), \tag{65.3}$$

$$\begin{aligned} -\alpha_{org}E_{\theta,org} = {} & -\left(\alpha^+_{MO}t^+_{MO\downarrow}E^+_{\theta,MO\downarrow}\chi^+ + \alpha^-_{MO}t^-_{MO\downarrow}E^-_{\theta,MO\downarrow}\chi^-\right) \\ & +\left(\alpha^+_{MO}t^+_{MO\uparrow}E^+_{\theta,MO\uparrow}\frac{1}{\chi^+} + \alpha^-_{MO}t^-_{MO\uparrow}E^-_{\theta,MO\uparrow}\frac{1}{\chi^-}\right), \end{aligned} \tag{65.4}$$

$$\begin{aligned} kE_{z,org} - i\alpha_{org}E_{r,org} = {} & k\left(t^+_{MO\downarrow}E^+_{z,MO\downarrow}\chi^+ + t^-_{MO\downarrow}E^-_{z,MO\downarrow}\chi^- + t^+_{MO\uparrow}E^+_{z,MO\uparrow}\frac{1}{\chi^+} + t^-_{MO\uparrow}E^-_{z,MO\uparrow}\frac{1}{\chi^-}\right) \\ & -i\alpha^+_{MO}\left(t^+_{MO\downarrow}\chi^+ - t^+_{MO\uparrow}\frac{1}{\chi^+}\right) - i\alpha^-_{MO}\left(t^-_{MO\downarrow}\chi^- - t^-_{MO\uparrow}\frac{1}{\chi^-}\right), \end{aligned} \tag{65.5}$$

$$E_{\theta,org} = t^+_{MO\downarrow}E^+_{\theta,MO\downarrow}\chi^+ + t^-_{MO\downarrow}E^-_{\theta,MO\downarrow}\chi^- + t^+_{MO\uparrow}E^+_{\theta,MO\uparrow}\frac{1}{\chi^+} + t^-_{MO\uparrow}E^-_{\theta,MO\uparrow}\frac{1}{\chi^-}. \tag{65.6}$$

Here, $\chi^\pm = e^{-\alpha^\pm_{MO}a}$ embodies the vertical thickness dependence of the problem. Supplementary Equations (64.1)–(65.6) can be manipulated to yield an entirely analogous expression to Supplementary Equation (22). This resulting expression can then be perturbatively expanded in g and the analogous procedure of section E of Supplementary Note 1 follows, with the caveat that the algebra becomes much more laborious. We bypass the latter by automatizing such work with Wolfram Mathematica(c). We summarize the results in the following sections of this Supplementary Note.

C. Perturbation expansion on g

1. Solving for $t^\pm_\downarrow$ and $t^\pm_\uparrow$

It is clear that provided that the frequencies Ω_0 and Ω_2 are modified accordingly (one finds $\Omega_1 = 0$ again), the expansions for the dielectric constants and the wavevectors can all be recycled from section E of Supplementary Note 1. On top of these quantities, following that section, we ought to expand α_{org} and $\chi^\pm$ up to $O(g^2)$ in Supplementary Equation (58),

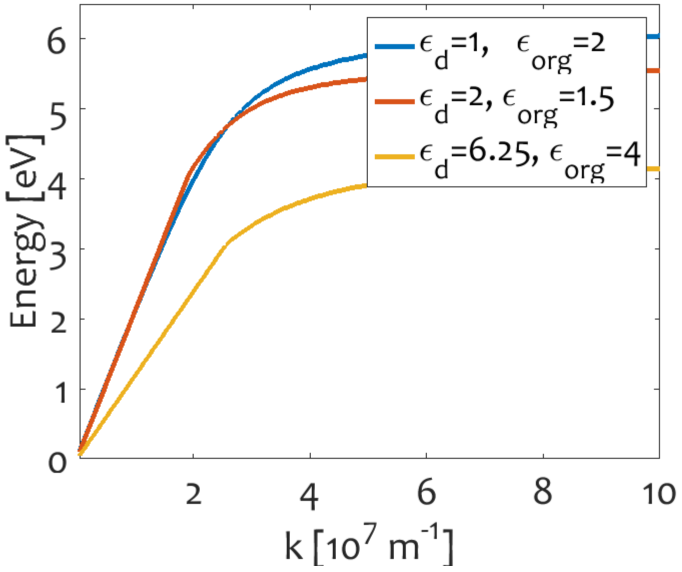

Supplementary Figure 4. *SP dispersion energy as a function of $k = |\boldsymbol{k}|$ for the three-layer (metal-MO dielectric-organic) setup, assuming $g = 0$.* Plots generated using Supplementary Equation (69) with Drude parameters for Ag, $\epsilon_\infty \sim 4$, $\omega_P \sim 9$ eV, and varying the dielectric permittivity ϵ_d as well as that of the organic layer. Since $\Omega = \Omega_0 + O(g^2)$, the plots are correct up to $O(g)$, which is our perturbation order of interest. Just like with Supplementary Figure (2), notice that the dispersion curves start-off linearly (light-like excitations), but plateau to constant values at large wavevectors (charge oscillations in the metal), which become smaller as the dielectric permittivity increases.

$$\alpha_{org} \approx \alpha_{org0} + g^2\alpha_{org22}\Omega_2, \tag{66.1}$$

$$\alpha_{org0} = \sqrt{k^2 - \Omega_0^2\epsilon_{org}}, \tag{66.2}$$

$$\alpha_{org22} = -\frac{\Omega_0\epsilon_{org}}{\alpha_{org0}}, \tag{66.3}$$

$$\chi^\pm \approx \chi_0\left\{1 \mp ag\alpha_{d1} - g^2\left[a^2\frac{\alpha_{d1}^2}{2} + a(\alpha_{d20} + \alpha_{d22}\Omega_2)\right]\right\}, \tag{67}$$

but we only need $t^\pm_{MO\downarrow}$ and $t^\pm_{MO\uparrow}$ up to $O(g)$,

$$t^\pm_{MO\downarrow} \approx t_{d\downarrow 0} \pm g t_{d\downarrow 1}, \tag{68.1}$$

$$t^\pm_{MO\uparrow} \approx t_{d\uparrow 0} \pm g t_{d\uparrow 1}. \tag{68.2}$$

Collecting the zeroth-order in g contributions in Supplementary Equations (64.1)–(65.6), we derive an implicit equation for Ω_0 which coincides with the standard textbook result for a three-layer system in the absence of an external magnetic field [14],

$$\chi_0^2 = \left(\frac{\alpha_{d0}\epsilon_{m0} + \alpha_{m0}\epsilon_d}{\alpha_{d0}\epsilon_{m0} - \alpha_{m0}\epsilon_d}\right)\left(\frac{\alpha_{d0}\epsilon_{org} + \alpha_{org0}\epsilon_d}{\alpha_{d0}\epsilon_{org} - \alpha_{org0}\epsilon_d}\right), \tag{69}$$

where α_{d0}, α_{m0}, α_{org0}, and ϵ_{m0} are all functions of Ω_0. This equation is the analogue of Supplementary Equations (27) and (28) for three layers. It is not possible to explicitly solve for Ω_0, but one can readily compute it numerically from such implicit equation (see Supplementary Figure 4). The formula for Ω_2 is too long to display it here and is, anyway, not relevant on its own. However, we make use of it to obtain our final results.

Next, we compute the coefficients in Supplementary Equations (68.1) and (68.2) in analogy to the two-layer case in Supplementary Equations (36) and (37). At zeroth-order in g,

$$t_{d\downarrow 0} = \frac{\alpha_{m0}\epsilon_d - \alpha_{d0}\epsilon_{m0}}{4\alpha_{m0}\epsilon_d}, \tag{70.1}$$

$$t_{d\uparrow 0} = \frac{\alpha_{m0}\epsilon_d + \alpha_{d0}\epsilon_{m0}}{4\alpha_{m0}\epsilon_d}, \tag{70.2}$$

while their first-order in g corrections are,

$$\begin{aligned} t_{d\downarrow 1} = \alpha_{d1}\Big(& a\alpha_{d0}^3(\chi_0^2-1)\epsilon_{m0} \\ & -\alpha_{d0}^2\{\chi_0^2[a\epsilon_{m0}(\alpha_{m0}+\alpha_{org0}) + a\alpha_{m0}\epsilon_d + \epsilon_{m0}] + a\alpha_{m0}\epsilon_d - a\alpha_{m0}\epsilon_{m0} + a\alpha_{org0}\epsilon_{m0} - \epsilon_{m0}\} \\ & +\alpha_{d0}\{a\alpha_{m0}^2(\chi_0^2+1)\epsilon_d + \alpha_{m0}(a\alpha_{org0}+1)[\chi_0^2(\epsilon_d+\epsilon_{m0}) - \epsilon_d + \epsilon_{m0}] + 2\alpha_{org0}\epsilon_{m0}\} \\ & -\alpha_{m0}^2(\chi_0^2-1)\epsilon_d(a\alpha_{org0}+1)\Big)/ \\ & \{4\alpha_{m0}\epsilon_d[\chi_0^2(\alpha_{m0}-\alpha_{d0})(\alpha_{d0}-\alpha_{org0}) + (\alpha_{d0}+\alpha_{m0})(\alpha_{d0}+\alpha_{org0})]\} \end{aligned} \tag{71.1}$$

and

$$\begin{aligned} t_{d\uparrow 1} = \Big[& \alpha_{d1}\chi_0^2\Big(\alpha_{d0}\epsilon_{m0}\{a\alpha_{d0}^2 - \alpha_{org0}[a(\alpha_{d0}+\alpha_{m0})+2] + a\alpha_{d0}\alpha_{m0} + \alpha_{d0} - \alpha_{m0}\} \\ & -\alpha_{m0}\epsilon_d(\alpha_{d0}+\alpha_{m0})[a(\alpha_{d0}-\alpha_{org0})-1]\Big) \\ & -\alpha_{d1}(\alpha_{d0}+\alpha_{m0})[a(\alpha_{d0}+\alpha_{org0})+1](\alpha_{d0}\epsilon_{m0}+\alpha_{m0}\epsilon_d)\Big]/ \\ & \{4\alpha_{m0}\epsilon_d[\chi_0^2(\alpha_{m0}-\alpha_{d0})(\alpha_{d0}-\alpha_{org0}) + (\alpha_{d0}+\alpha_{m0})(\alpha_{d0}+\alpha_{org0})]\}. \end{aligned} \tag{71.2}$$

Hence, at zeroth order, $t^+_{MO\downarrow} + t^-_{MO\downarrow} = 2t_{d\downarrow 0}$ and $t^+_{MO\uparrow} + t^-_{MO\uparrow} = 2t_{d\uparrow 0}$, and these total coefficients for exponentially decreasing and increasing fields become identical to the textbook results for SP modes in the three-layer setup in the absence of an external magnetic field.

2. Collecting the expressions for the fields

For reference, the zeroth-order fields are given by,

$$\vec{E}_{org0} = (E_{k,org0}, 0, E_{z,org0})\eta_0 e^{-\alpha_{org0}z}, \tag{72.1}$$

$$\vec{B}_{org0} = (0, B_{\theta,org0}, 0)\eta_0 e^{-\alpha_{org0}z}, \tag{72.2}$$

$$\vec{E}_{d0} = 2t_{d\downarrow 0}(1, 0, E_{z,d\downarrow 0})\eta_0 e^{-\alpha_{d0}z} + 2t_{d\uparrow 0}(1, 0, E_{z,d\uparrow 0})\eta_0 e^{\alpha_{d0}z}, \tag{72.3}$$

$$\vec{B}_{d0} = 2t_{d\downarrow 0}(0, B_{\theta,d\downarrow 0}, 0)\eta_0 e^{-\alpha_{d0}z} + 2t_{d\uparrow 0}(0, B_{\theta,d\uparrow 0}, 0)\eta_0 e^{\alpha_{d0}z}, \tag{72.4}$$

$$\vec{E}_{m0} = (1, 0, E_{z,m0})\eta_0 e^{-\alpha_{m0}z}, \tag{72.5}$$

$$\vec{B}_{m0} = (0, B_{\theta,m0}, 0)\eta_0 e^{-\alpha_{m0}z}, \tag{72.6}$$

with each of the components being,

$$E_{k,org0} = \frac{\alpha_{m0}(\chi_0^2+1)\epsilon_d - \alpha_{d0}(\chi_0^2-1)\epsilon_{m0}}{2\alpha_{m0}\chi_0\epsilon_d}, \tag{73.1}$$

$$E_{z,org0} = -\frac{ik[\alpha_{d0}(\chi_0^2+1)\epsilon_{m0} + \alpha_{m0}(\epsilon_d - \chi_0^2\epsilon_d)]}{2\alpha_{d0}\alpha_{m0}\chi_0\epsilon_{org}}, \tag{73.2}$$

$$B_{\theta,org0} = \frac{i\{2\alpha_{d0}\alpha_{m0}\alpha_{org0}\chi_0\epsilon_{org} + k^2[\alpha_{d0}(\chi_0^2+1)\epsilon_{m0} + \alpha_{m0}(\epsilon_d - \chi_0^2\epsilon_d)]\}}{2\alpha_{d0}\alpha_{m0}\chi_0\Omega_0 c\epsilon_{org}}, \tag{73.3}$$

$$E_{z,d\downarrow 0} = -E_{z,d\uparrow 0} = \frac{ik}{\alpha_{d0}}, \tag{73.4}$$

$$B_{\theta,d\downarrow 0} = -B_{\theta,d\uparrow 0} = -\frac{i(k^2-\alpha_{d0}^2)}{\alpha_{d0}\Omega_0 c}, \tag{73.5}$$

$$E_{z,m0} = -\frac{ik}{\alpha_{m0}}, \tag{73.6}$$

$$B_{\theta,m0} = \frac{i(k^2-\alpha_{m0}^2)}{\alpha_{m0}\Omega_0 c}. \tag{73.7}$$

The expressions for the $O(g)$ fields at each layer are also cumbersome; we only show the electric field in the organic layer, as it is the one associated with the coupling with excitons (see Supplementary Equation (39.1)),

$$\vec{E}_{org} \approx \vec{E}_{org0} + g\vec{E}_{org1}. \tag{74}$$

The first order in g contribution is

$$\vec{E}_{org1} = \boldsymbol{E}_{org1}\eta_0 e^{-\alpha_{org0}z}, \tag{75}$$

where $\boldsymbol{E}_{org1}$ is purely tangential and purely-imaginary valued,

$$\begin{aligned}\boldsymbol{E}_{org1}\cdot\hat{\boldsymbol{\theta}} = -i\Omega_0^2\Big(&\alpha_{m0}\epsilon_d\{a[\chi_0^2(\alpha_{d0}-\alpha_{org0}) + \alpha_{d0} + \alpha_{org0}] - \chi_0^2 + 1\} \\ &+\epsilon_{m0}\{\chi_0^2[a\alpha_{d0}(\alpha_{org0}-\alpha_{d0}) + \alpha_{org0}] + a\alpha_{d0}(\alpha_{d0}+\alpha_{org0}) - \alpha_{org0}\}\Big)/ \\ &2\alpha_{m0}\epsilon_d[\chi_0^2(\alpha_{m0}-\alpha_{d0})(\alpha_{d0}-\alpha_{org0}) + (\alpha_{d0}+\alpha_{m0})(\alpha_{d0}+\alpha_{org0})].\end{aligned} \tag{76}$$

Compared with Supplementary Equation (41.1), the z-dependence of $\vec{E}_{org1}$ is purely exponential, as the lowest order correction of α_{org} to α_{org0} is $O(g^2)$.

D. Quantization and normalization of modes

Equipped with these results, we use Supplementary Equations (64.1)–(65.6) to compile expressions for the fields in each layer. First, we aim to compute the vertical normalization length $L_{\boldsymbol{k}}$ for each mode. In analogy to Supplementary Equation (50), we obtain,

$$\begin{aligned}L_{\boldsymbol{k}} = &\sum_i \left[\epsilon_0\epsilon_{org}|E_{i,org}|^2 + \frac{1}{\mu_0\mu}|B_{i,org}|^2\right]\frac{1}{\epsilon_0}\int_a^\infty dz e^{-(\alpha_{org}^*+\alpha_{org})z} \\ &+\sum_{ij}\sum_{\gamma,\delta\in\{+,-\}}\sum_{u,v\in\{\uparrow,\downarrow\}}(t_{MOu}^{\gamma})^* t_{MOv}^{\delta}\left[\epsilon_0(\epsilon_{MO,ij}E_{j,MO}^{\gamma})^* E_{i,MO}^{\delta} + \frac{1}{\mu_0\mu}(B_{i,MO}^{\gamma})^* B_{i,MO}^{\delta}\right] \\ &\times\frac{1}{\epsilon_0}\int_0^a dz e^{[(\mathrm{sgn}(u)(\alpha_{MO}^{\gamma})^* + \mathrm{sgn}(v)\alpha_{MO}^{\delta}]z} \\ &+\sum_i\left[\epsilon_0\frac{d(\omega\epsilon_m(\omega))}{d\omega}|E_{i,m}|^2 + \frac{1}{\mu_0\mu}|B_{i,m}|^2\right]\frac{1}{\epsilon_0}\int_{-\infty}^0 dz e^{(\alpha_m^*+\alpha_m)z},\end{aligned} \tag{77}$$

where sgn(↑) = −sgn(↓) = 1 denote the exponentially increasing or decreasing fields, respectively. At $O(g)$, $L_{\boldsymbol{k}} \approx L_{\boldsymbol{k}0}$ as in Supplementary Equation (56), and the anisotropy of the MO layer is unimportant. Carrying out the integrations explicitly (and taking care of the possibility that α_{d0} is complex-valued),

$$\begin{aligned} L_{\boldsymbol{k}0} = &\sum_i \left[\epsilon_0\epsilon_{org}|E_{i,org0}|^2 + \frac{1}{\mu_0\mu}|B_{i,org0}|^2\right]\frac{e^{-2\Re\alpha_{org0}a}}{2\epsilon_0\Re\alpha_{org0}} \\ &+4\sum_i \left|t_{d\uparrow 0}\right|^2\left[\epsilon_0\epsilon_d|E_{i,d\uparrow 0}|^2 + \frac{1}{\mu_0\mu}|B_{i,d\uparrow 0}|^2\right]\left(\frac{e^{2\Re\alpha_{d0}a}-1}{2\epsilon_0\Re\alpha_{d0}}\right) \\ &+4\sum_i \left|t_{d\downarrow 0}\right|^2\left[\epsilon_0\epsilon_d|E_{i,d\downarrow 0}|^2 + \frac{1}{\mu_0\mu}|B_{i,d\downarrow 0}|^2\right]\left(\frac{1-e^{-2\Re\alpha_{d0}a}}{2\epsilon_0\Re\alpha_{d0}}\right) \\ &+\left\{4\sum_{ij}(t_{d\uparrow 0})^*(t_{d\downarrow 0})\left[\epsilon_0\sum_i\epsilon_d(E_{i,d\uparrow 0})^*E_{i,d\downarrow 0} + \frac{1}{\mu_0\mu}(B_{i,d\uparrow 0})^*B_{i,d\downarrow 0}\right]\left(\frac{a}{\epsilon_0}\right) + \text{c.c.}\right\} \\ &+\sum_i\left[\epsilon_0\frac{d(\omega\epsilon_m(\omega))}{d\omega}|E_{i,m0}|^2 + \frac{1}{\mu_0\mu}|B_{i,m0}|^2\right]\frac{1}{2\epsilon_0\Re\alpha_{m0}}, \end{aligned} \tag{78}$$

One may numerically compute $L_{\boldsymbol{k}}$ by plugging Supplementary Equations (70.1), (70.2), and (72.1)–(73.7) into Supplementary Equation (78). We do not display the resulting analytical expression, which anyhow, is lengthy and not particularly illuminating.

Supplementary Note 3. EXCITON-EXCITON AND EXCITON-SP COUPLINGS

In Secs. Supplementary Note 1 and Supplementary Note 2, we solved for the electromagnetic profile of the magneto-SP modes in a two- and three-layer setup. We are now ready to describe the organic superlattice. We regard the latter to be either "embedded" in the MO layer (in the two layer setup) or in its separate third layer (in the three-layer one). As explained in the following paragraphs, the superlattice consists of a monoclinic array of organic aggregate nanopillars. For simplicity, we take each of the nanopillars to be a rectangular parallelepiped of volume $W_x W_y W_z$ (here, W_i is the width of the nanopillar along the i-th axis). If the nanopillar density is ρ_{np}, it contains $N_{np} = \rho_{np} W_x W_y W_z$ chromophores. Furthermore, the three-dimensional positions of the individual chromophores constituting each nanopillar are denoted,

$$\boldsymbol{r}_{\boldsymbol{m}s} = \underbrace{(m_x\delta_x\hat{\boldsymbol{x}} + m_y\delta_y\hat{\boldsymbol{y}})}_{\equiv \boldsymbol{r}_{\boldsymbol{m}}} + \underbrace{s\delta_z}_{\equiv z_s}\hat{\boldsymbol{z}}, \tag{79}$$

where δ_i is the spacing between chromophores along the i-th direction,

A. Dipolar couplings between nanopillars

We shall first study the energetic contribution due to the excitons alone. We model this as,

$$H_{exc} = \sum_{\boldsymbol{n},s} \omega_{\boldsymbol{n}} \sigma_{\boldsymbol{n}}^\dagger \sigma_{\boldsymbol{n}} + \sum_{\boldsymbol{n}\neq\boldsymbol{n}'} (J_{\boldsymbol{n}\boldsymbol{n}'} \sigma_{\boldsymbol{n}}^\dagger \sigma_{\boldsymbol{n}'} + \text{h.c.}), \tag{80}$$

where $\omega_{\boldsymbol{n}}$ is the bare energy of the $\boldsymbol{n}$-th collective nanopillar dipole, which we take in the ideal case to be $\omega_{\boldsymbol{n}} = \bar{\omega}$. The exciton hopping amplitude between the $\boldsymbol{n}$-th and $\boldsymbol{n}'$-th nanopillars is approximated as a near-field dipolar coupling,

$$J_{\boldsymbol{n}\boldsymbol{n}'} = \frac{\eta}{\epsilon|\boldsymbol{r}_{\boldsymbol{n}} - \boldsymbol{r}_{\boldsymbol{n}'}|^3}\left[\boldsymbol{\mu}_{\boldsymbol{n}}\cdot\boldsymbol{\mu}_{\boldsymbol{n}'} - 3(\boldsymbol{\mu}_{\boldsymbol{n}}\cdot\boldsymbol{e}_{\boldsymbol{n}\boldsymbol{n}'})(\boldsymbol{\mu}_{\boldsymbol{n}'}\cdot\boldsymbol{e}_{\boldsymbol{n}\boldsymbol{n}'})\right], \tag{81}$$

where $\eta = 0.625\,\text{meV}(\text{nm}^3/\text{D}^2)$, $\boldsymbol{e}_{\boldsymbol{n}\boldsymbol{n}'} = \frac{\boldsymbol{r}_{\boldsymbol{n}} - \boldsymbol{r}_{\boldsymbol{n}'}}{|\boldsymbol{r}_{\boldsymbol{n}} - \boldsymbol{r}_{\boldsymbol{n}'}|}$, where $\boldsymbol{r}_{\boldsymbol{n}}$ is the average in-plane location of the $\boldsymbol{n}$-th nanopillar, and we take $\epsilon \approx 1$, the dielectric permittivity in the medium surrounding the nanopillars. Supplementary Equation (81) implicitly relies on a separation of energy scales, namely, that the coupling between chromophores is much stronger within a single nanopillar than between different ones. Hence, we start with the collective superradiant nanopillar transitions which scale as $\boldsymbol{\mu}_{\boldsymbol{n}} = \sum_{\boldsymbol{m}s} \boldsymbol{p}_{\boldsymbol{m}s} \approx \sqrt{N_{np}}\boldsymbol{p}_{\boldsymbol{n}}$ [17], where $\boldsymbol{p}_{\boldsymbol{m}s}$ is the transition dipole moment of the $\boldsymbol{m}s$-th chromophore in the nanopillar, the sum is over all $\boldsymbol{m}s$ values associated with the $\boldsymbol{n}$-th nanopillar, and $\boldsymbol{p}_{\boldsymbol{m}s} = \boldsymbol{p}_{\boldsymbol{n}}$, that is, we take the dipole to be equal for all chromophores within the corresponding nanopillar. This approximation should provide a semi-quantitative description of the dispersion of the organic superlattice alone. A more refined description would rely on the coupled-dipole method [18–20], but is beyond the scope of this work, as this simplified model illustrates the essence of the problem.

We shall now consider a general two-dimensional monoclinic superlattice with unit cell defined by vectors $\vec{OD}$ and $\vec{OC}$ depicted in Supplementary Figure 5. For convenience, we temporarily adopt the θ-rotated coordinate system $x'y'$ which, with respect to the original xy system, is defined by,

$$\begin{bmatrix} \hat{\boldsymbol{x}} \\ \hat{\boldsymbol{y}} \end{bmatrix} = \begin{bmatrix} \cos\theta & -\sin\theta \\ \sin\theta & \cos\theta \end{bmatrix} \begin{bmatrix} \hat{\boldsymbol{x}}' \\ \hat{\boldsymbol{y}}' \end{bmatrix}. \tag{82}$$

We will later explain how to obtain θ. We take two sides of the parallelogram (AB and CD) to be parallel to $\hat{\boldsymbol{x}}'$. For simplicity, the nanopillars are taken to be rectangular parallelepipeds. Their transition dipoles $\boldsymbol{\mu}_{\boldsymbol{n}} = \boldsymbol{\mu}$ are fixed in the $x'y'$ plane and make an angle α' with respect to $\hat{\boldsymbol{x}}'$ (or $\alpha \equiv \alpha' + \theta$ with respect to $\hat{\boldsymbol{x}}$). Notice that all sites are equivalent. We only account for nearest-neighbor (NN) and next-nearest-neighbor interactions (NNN). We classify the interactions as horizontal NNN (AB, CD), vertical NNN (AD, BC), diagonal type A NN (OA, OC), and diagonal type B NN (OB,OD), respectively,

$$J_h = \eta\mu^2 \frac{(1-3\cos^2\alpha')}{\Delta_h^3}, \tag{83.1}$$

$$J_v = \eta\mu^2 \frac{[1-3\cos^2(\alpha'-\beta)]}{\Delta_v^3}, \tag{83.2}$$

$$J_{diagA} = \eta\mu^2 \frac{[1-3\cos^2(\alpha'-\gamma)]}{\left(\frac{\Delta_h^2+2\Delta_h\Delta_v\cos\beta+\Delta_v^2}{4}\right)^{3/2}}, \tag{83.3}$$

$$J_{diagB} = \eta\mu^2 \frac{[1-3\cos^2(\alpha'+\delta)]}{\left(\frac{\Delta_h^2-2\Delta_h\Delta_v\cos\beta+\Delta_v^2}{4}\right)^{3/2}}. \tag{83.4}$$

Here, $|\boldsymbol{\mu}| = \mu$ and the side lengths are $\overline{AB} = \overline{CD} = \Delta_h$ and $\overline{BC} = \overline{AD} = \Delta_v$. We have also conveniently introduced the angles $\beta \equiv \angle BCD = \angle DAB$, as well as the following,

$$\gamma = \operatorname{atan}\frac{\Delta_v\sin\beta}{\Delta_h + \Delta_v\cos\beta}, \tag{84.1}$$

$$\delta = \operatorname{atan}\frac{\Delta_v\sin\beta}{\Delta_h - \Delta_v\cos\beta}. \tag{84.2}$$

Assuming that all site energies are equal, $\omega_{\boldsymbol{n}} = \bar{\omega}$, we may rewrite Supplementary Equation (80) in $\boldsymbol{k}$ space, $H_{exc} = \sum_{\boldsymbol{k}} H_{exc,\boldsymbol{k}}$, where $H_{exc,\boldsymbol{k}} = \omega_{exc,\boldsymbol{k}}\sigma^\dagger_{\boldsymbol{k}}\sigma_{\boldsymbol{k}}$, where $\boldsymbol{k} = k_{x'}\hat{\boldsymbol{x}}' + k_{y'}\hat{\boldsymbol{y}}'$ and

$$\begin{aligned}\omega_{exc,\boldsymbol{k}} = \bar{\omega} &+ 2J_h\cos(k_{x'}\Delta_h) + 2J_v\cos\left[k_{x'}\Delta_v\cos\beta + k_{y'}\Delta_v\sin\beta\right] \\ &+2J_{diagA}\cos\left[k_{x'}\frac{\Delta_h+\Delta_v\cos\beta}{2} + k_{y'}\frac{\Delta_v\sin\beta}{2}\right] \\ &+2J_{diagB}\cos\left[k_{x'}\frac{\Delta_h-\Delta_v\cos\beta}{2} - k_{y'}\frac{\Delta_v\sin\beta}{2}\right]\end{aligned} \tag{85}$$

is the resulting dispersion relation for the excitons alone.

As explained in the main text, we would ideally like to design a plexciton dispersion which features a global gap in the bulk. This requires a superlattice with an "H-aggregate" dispersion along all wavevector directions. Mathematically, this means that the dispersion $\omega_{exc,\boldsymbol{k}}$ should be a maximum at $\boldsymbol{k} = 0$. It turns out that this is not possible in a rectangular lattice ($\beta = \frac{\pi}{2}$), as the resulting J-couplings ($J_i < 0$) arising from the geometric constraints end up dominating the H-couplings ($J_i > 0$) at least along one direction, yielding a minimum, or at best a saddle point for $\omega_{exc,\boldsymbol{k}}$ at $\boldsymbol{k} = 0$. Hence, we proceed in a more systematic fashion. Taylor expanding Supplementary Equation (85) up to quadratic order in $k_{i'}$,

$$\omega_{exc,\boldsymbol{k}} \approx \bar{\omega}'_{eff} + \begin{bmatrix} k_{x'} & k_{y'} \end{bmatrix}\begin{bmatrix} M_{x'x'} & M_{x'y'} \\ M_{y'x'} & M_{y'y'}\end{bmatrix}\begin{bmatrix} k_{x'} \\ k_{y'}\end{bmatrix}, \tag{86}$$

where the constant offset is obtained by evaluating $\omega_{exc,\boldsymbol{k}}$ at $\boldsymbol{k} = 0$,

$$\bar{\omega}'_{eff} = \bar{\omega} + 2J_h + 2J_v + 2J_{diagA} + 2J_{diagB}. \tag{87}$$

Here, $M_{i'j'} = \frac{1}{2}\frac{\partial^2 E_{exc,\boldsymbol{k}}}{\partial k_{i'}\partial k_{j'}}\Big|_{\boldsymbol{k}=0}$ denotes a Hessian matrix, which can be readily diagonalized,

$$\begin{bmatrix} M_{x'x'} & M_{x'y'} \\ M_{y'x'} & M_{y'y'}\end{bmatrix} = \begin{bmatrix} S_{xx'} & S_{yx'} \\ S_{xy'} & S_{yy'}\end{bmatrix}\begin{bmatrix} m_x & 0 \\ 0 & m_y\end{bmatrix}\begin{bmatrix} S_{xx'} & S_{xy'} \\ S_{yx'} & S_{yy'}\end{bmatrix}. \tag{88}$$

where $S_{ij'}$ is a unitary matrix, and ultimately yields the result,

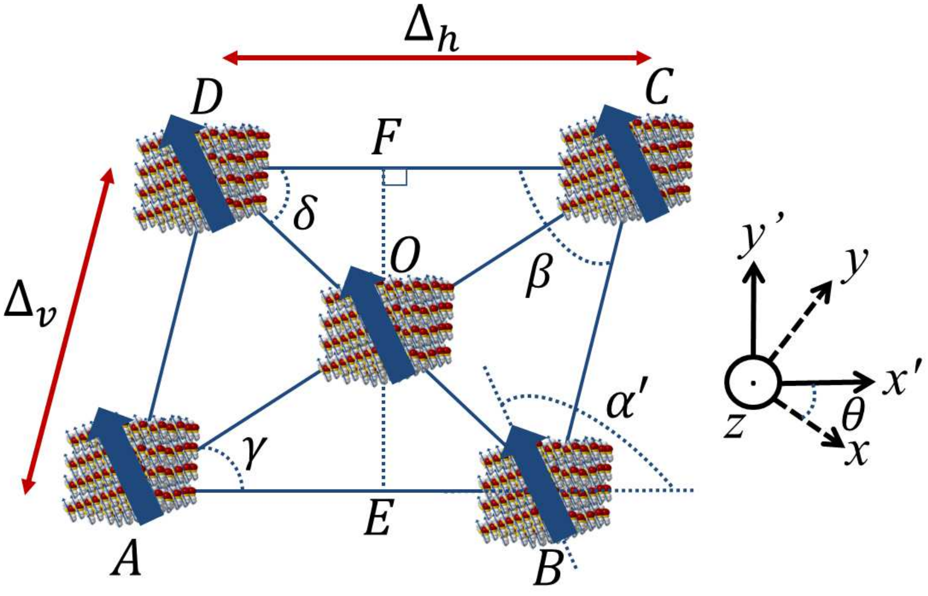

Supplementary Figure 5. *Geometry of two-dimensional monoclinic superlattice of organic nanopillars.* $x'y'$ denotes a temporary Cartesian coordinate system which is rotated at θ from the original xy system. The collective transition dipole moments of the nanopillars make an angle α' with respect to $\hat{\boldsymbol{x}}'$ (or $\alpha \equiv \alpha' + \theta$ with respect to $\hat{\boldsymbol{x}}$). The horizontal and vertical distances Δ_h and Δ_v, together with the angles β, γ, and δ fully define the superlattice.

$$\omega_{exc,\boldsymbol{k}} \approx \bar{\omega}'_{eff} + m_x k_x^2 + m_y k_y^2 \tag{89.1}$$

$$\approx \bar{\omega}_{eff} + 2J_x \cos k_x \Delta_x + 2J_y \cos k_y \Delta_y, \tag{89.2}$$

where $k_i = S_{ix'} k_{x'} + S_{iy'} k_{y'}$, and for our simulation, we choose (arbitrary) effective unit cell dimensions Δ_i such that $J_i = -\frac{m_i}{\Delta_i^2}$ for $i = x, y$, and $\bar{\omega}'_{eff} = \bar{\omega}_{eff} + 2J_x + 2J_y$; this identification renders Supplementary Equations (89.1) and (89.2) equal up to quadratic order in k_i. We have thus arrived at a very convenient expression; Supplementary Equation (89.2) shows that the oblique lattice renders the same long-wavelength physics as a much simpler rectangular lattice with only NN interactions. This approximation is insightful in that it exposes the physical origin of the global gap; it also remains valid for our purposes as the topological phenomena of our interest occurs at small k_i.

For an arbitrary fixed value of Δ_h, we Monte Carlo sample through the parameters $r \equiv \frac{\Delta_v}{\Delta_h} \in [0,2]$, $\alpha \in [0,\pi]$, and $\beta \in [0,\pi]$ and record those which yield $m_x, m_y < 0$. We observe that only ~8% of the parameter space satisfies the H-aggregate condition we are looking for. One such set of parameters is $\alpha' = 1.20(= 68.5\,\text{degrees})$, $\beta = 0.23(= 13.1\,\text{degrees})$, $r = 0.88$, yielding $(m_x, m_y) = -(1.49, 0.44) J_0 \Delta_h^2$, where $J_0 \equiv \frac{\eta\mu^2}{\Delta_h^3}$ sets the energy scale of the dipolar interactions. The associated eigenvector matrix is,

$$\begin{bmatrix} S_{xx'} & S_{yx'} \\ S_{xy'} & S_{yy'} \end{bmatrix} = \begin{bmatrix} -0.91 & -0.41 \\ 0.41 & -0.91 \end{bmatrix} = \begin{bmatrix} \cos\theta & -\sin\theta \\ \sin\theta & \cos\theta \end{bmatrix} \tag{90}$$

where we obtain $\theta = 2.72(= 155.7^o)$, which defines the angle of rotation of our temporary coordinates $x'y'$ with respect to the original ones xy; then, $\alpha = \alpha' + \theta = 3.9(= 224^o)$. Now, each nanopillar has a collective transition dipole moment value of $\mu = \sqrt{N_{np}}|\boldsymbol{p_n}| = \sqrt{N_{np}} \times 12\,\text{D} = \sqrt{W_x W_y W_z \rho_{np}} \times 12\,\text{D}$ (D=Debye). Choosing the nanopillars to be separated from one another by Δ_h, we Nget a value for the energy scale of $J_0 = \frac{\eta W_x W_y W_z \rho_{np}}{\Delta_h^3} \times 100\,\text{D}^2$. Taking $\rho_{np} = 38\,\text{molecules/nm}^3$, we obtain,

$$J_x = -\frac{m_x}{\Delta_x^2} = 3.45\,\text{eV} \times \zeta_x$$
$$J_y = -\frac{m_y}{\Delta_y^2} = 1.02\,\text{eV} \times \zeta_y$$

where $\zeta_i = \frac{W_x W_y W_z}{\Delta_h \Delta_i^2}$ are dimensionless ratios which govern the effective dispersion of the superlattice. By choosing the physically reasonable parameters $\Delta_h = 100\,\text{nm}$, $W_x = 10\,\text{nm}$, $W_y = 75\,\text{nm}$, $W_z = 70\,\text{nm}$, $\bar{\omega}_{eff} = 0.57\,\text{eV}$ (the latter is chosen to obtain maximum coupling of the exciton with the magneto-optical interaction) and the effective simulation parameters $\Delta_x = \Delta_y = 50\,\text{nm}$, we obtain $\Delta_v = r\Delta_h = 88\,\text{nm}$, $\zeta_x = \zeta_y = 0.21$, $N_{np} = 2\times 10^6$ molecules, $\mu = 1.7\times 10^4\,\text{D}$, $J_x = 1.04\,\text{eV}$, $J_y = 0.31\,\text{eV}$. We emphasize that even though J_x and J_y can have arbitrary values which depend on our choice of parameters Δ_x and Δ_y, the latter set the spatial resolution of our real space simulations, and hence, the size of the systems we can computationally study. These simulations are carried out in order to calculate edge states as well as understand the effects of disorder.

We note that when choosing parameters, we need to make sure that (a) the nanopillars do not yuxtapose each other and (b) the number of chromophores in the organic layer is large enough to achieve considerable coupling with the SPs (see Supplementary Equations (96) and (99)), (c) the thickness W_z is not too large to induce very strong dipolar interactions between nanopillars which overwhelm the exciton-SP coupling in (b). Condition (a) is easily checked computationally and graphically. With respect to condition (b), we note that the surface area of the $ABCD$ parallelogram in Supplementary Figure 5 is $\Delta_h \Delta_y \sin\beta$. It contains two nanopillars of surface area $W_x W_y$. The surface coverage fraction of the organic layer is hence,

$$f = \frac{2W_x W_y}{\Delta_h \Delta_v \sin\beta}. \tag{91}$$

For our chosen parameters, $f = 0.78$. In general, we need both f and W_z to not be very small ($f > 0.2$, $W_z > 40\,\text{nm}$) in order to achieve strong exciton-plasmon coupling. Condition (c) is optimized numerically and yields parameters $W_z < 100\,\text{nm}$.

In the next section of this Supplementary Note, we shall work with the effective rectangular superlattice of $N_x \times N_y$ nanopillars (where N_i is the number of nanopillars along the i-th direction) instead of the original monoclinic one. This is a good approximation not only for the interactions between the various nanopillars, but also for the exciton-SP couplings, as long we use the average density of the original monoclinic lattice (see Supplementary Equations (96) and (99)).

B. Exciton-SP couplings

We are now ready to discuss the effective interaction between SPs and a single nanopillar. Consider the dipole operator $\hat{\boldsymbol{p}}_{\boldsymbol{m}s} = \boldsymbol{p}_{\boldsymbol{m}s}(b^\dagger_{\boldsymbol{m}s} + b_{\boldsymbol{m}s})$, where $b^\dagger_{\boldsymbol{m}s}(b_{\boldsymbol{m}s})$ creates (annhilates) an exciton at the $\boldsymbol{m}s$-th chromophore of some nanopillar. The time-independent electric field operator is $\hat{\vec{\mathscr{E}}}'(\boldsymbol{r}) \equiv \sum_{\boldsymbol{k}} \sqrt{\frac{\omega(\boldsymbol{k})}{2\epsilon_0 S L_{\boldsymbol{k}}}} a_{\boldsymbol{k}} \vec{E}'(\boldsymbol{k}) + \text{h.c.}$, which results from transforming Supplementary Equation (55.1) from the Heisenberg to the Schrodinger picture by removing the dynamical phases $e^{-i\omega(\boldsymbol{k})t}$ (see Supplementary Equations (18.1) and (20.1)), i.e., $\vec{E}'(\boldsymbol{k}) \equiv \vec{E}(\boldsymbol{k}) e^{i\omega(\boldsymbol{k})t}$. Using Supplementary Equation (74) and (75), the dipolar coupling between the $\boldsymbol{n}$-th nanopillar and the SP modes is given by

$$\begin{aligned} H^{(\boldsymbol{n})}_{exc-SP} &= -\sum_{\boldsymbol{m},s} \hat{\boldsymbol{p}}_{\boldsymbol{m}s} \cdot \hat{\vec{\mathscr{E}}}'(\boldsymbol{r}) \\ &= \sum_{\boldsymbol{k},\boldsymbol{m},s} \mathscr{J}_{\boldsymbol{k},\boldsymbol{m}s} a_{\boldsymbol{k}} b^\dagger_{\boldsymbol{m}s} e^{i\boldsymbol{k}\cdot\boldsymbol{r}_{\boldsymbol{m}}} + \text{h.c.}, \end{aligned} \tag{92}$$

where the sum over $\boldsymbol{m}s$ is restricted to the chromophores in the $\boldsymbol{n}$-th nanopillar. We have also used the rotating-wave approximation to discard far-off-resonant terms of the form $a_{\boldsymbol{k}}\sigma_{\boldsymbol{n}s}$ and $a^\dagger_{\boldsymbol{k}}\sigma^\dagger_{\boldsymbol{n}s}$. Using Supplementary Equation (55.1), the corresponding coupling is given by,

$$\mathscr{J}_{\boldsymbol{k},\boldsymbol{m}s} = -\sqrt{\frac{\omega(\boldsymbol{k})}{2\epsilon_0 S L_{\boldsymbol{k}}}} e^{-\alpha(\boldsymbol{k})z_s} \boldsymbol{p}_{\boldsymbol{m}s} \cdot \boldsymbol{E}(\boldsymbol{k}, z_s), \tag{93}$$

where, depending on whether we use the two-layer or three-layer setup results, we make the following substitutions,

Two-layer setup		
$L_{\boldsymbol{k}}$	$L_{\boldsymbol{k}0}$	Supplementary Equation (50)
α	α_{d0}	Supplementary Equation (24.2)
$\boldsymbol{E}(\boldsymbol{k},z_s)$	$\boldsymbol{E}_{MO}(\boldsymbol{k},z_s)=\boldsymbol{E}_{d0}(\boldsymbol{k})+g\boldsymbol{E}_{d1}(\boldsymbol{k},z_s)$	Supplementary Equations (40.1), (41.1)

Three-layer setup		
$L_{\boldsymbol{k}}$	$L_{\boldsymbol{k}0}$	Supplementary Equation (78)
α	α_{org0}	Supplementary Equation (66.2)
$\boldsymbol{E}(\boldsymbol{k},z_s)$	$\boldsymbol{E}_{org}(\boldsymbol{k},z_s)=\boldsymbol{E}_{org0}(\boldsymbol{k})+g\boldsymbol{E}_{org1}(\boldsymbol{k},z_s)$	Supplementary Equations (72.1), (73.1), (73.2), (76).

Supplementary Equation (93) exposes the 3D nature of our problem with its z_s dependence: there is an exponential contribution $e^{-\alpha(\boldsymbol{k})z_s}$ from the SP evanescent field, and even a linear correction in z_s due to $\boldsymbol{E}_{d1}$ for the two-layer setup. In any case, it will prove convenient to derive an effective 2D description for our model. We have two ways to do so.

1. *Mean-field approximation (MFA)*

Using Supplementary Equations (92) and (93),

$$H^{(\boldsymbol{n})}_{exc-SP}=-\sum_{\boldsymbol{k}}\sqrt{\frac{\omega(\boldsymbol{k})}{2\epsilon_0 SL_{\boldsymbol{k}}}}e^{-\alpha(\boldsymbol{k})\bar{z}(\boldsymbol{k})}a_{\boldsymbol{k}}\boldsymbol{p}_{\boldsymbol{m}s}\cdot\left[\sum_{\boldsymbol{m}s}\boldsymbol{E}(\boldsymbol{k},z_s)e^{i\boldsymbol{k}\cdot(\boldsymbol{r}_{\boldsymbol{m}}-\boldsymbol{r}_{\boldsymbol{n}})-\alpha_0(\boldsymbol{k})(z_s-\bar{z}(\boldsymbol{k}))}b^{\dagger}_{\boldsymbol{m}s}\right]e^{i\boldsymbol{k}\cdot\boldsymbol{r}_{\boldsymbol{n}}}+\text{c.c.} \tag{94.1}$$

$$\approx-\sum_{\boldsymbol{k}}\underbrace{\sqrt{\frac{\omega(\boldsymbol{k})}{2\epsilon_0 SL_{\boldsymbol{k}}}}e^{-\alpha(\boldsymbol{k})\bar{z}(\boldsymbol{k})}e^{i\boldsymbol{k}\cdot\boldsymbol{r}_{\boldsymbol{n}}}\boldsymbol{\mu}_{\boldsymbol{n}}\cdot\boldsymbol{E}(\boldsymbol{k},\bar{z}(\boldsymbol{k}))}_{\equiv\mathscr{J}_{\boldsymbol{kn}}}a_{\boldsymbol{k}}\sigma^{\dagger}_{\boldsymbol{n}}+\text{c.c.}, \tag{94.2}$$

where we have formally taken $\bar{z}(\boldsymbol{k})$ to be an average ($\boldsymbol{k}$-dependent) vertical position for the chromophores in the nanopillar (we will discuss how to compute this parameter later, see Supplementary Equation (101)), made the MFA that $e^{i\boldsymbol{k}\cdot(\boldsymbol{r}_{\boldsymbol{m}}-\bar{\boldsymbol{r}})-\alpha_0(\boldsymbol{k})(z_s-\bar{z})}\approx 1$, assumed that the dipoles $\boldsymbol{p}_{\boldsymbol{m}s}=\boldsymbol{p}_{\boldsymbol{n}}$ for all the chromophores in the $\boldsymbol{n}$-th nanopillar, and defined the collective exciton operator,

$$\sigma^{\dagger}_{\boldsymbol{n}}=\frac{1}{\sqrt{N_{np}}}\sum_{\boldsymbol{m}s}b^{\dagger}_{\boldsymbol{m}s}, \tag{95}$$

such that its corresponding transition dipole is superradiantly enhanced at $\boldsymbol{\mu}_{\boldsymbol{n}}=\langle 0|\sigma_{\boldsymbol{n}}\sum_{\boldsymbol{m}s}\hat{\boldsymbol{p}}_{\boldsymbol{m}s}|0\rangle=\sqrt{N_{np}}\boldsymbol{p}_{\boldsymbol{n}}$ [17].

Having addressed the effective interaction between a single nanopillar and the SP modes, we can move on to the description of the superlattice, $H_{exc-SP}=\sum_{\boldsymbol{n}}H^{(\boldsymbol{n})}_{exc-SP}$. If $\boldsymbol{\mu}_{\boldsymbol{n}}=\boldsymbol{\mu}$ and we assume periodic boundary conditions (PBCs), we can construct Fourier $\boldsymbol{k}$ modes for the excitons too, $\sigma^{\dagger}_{\boldsymbol{k}}=\frac{1}{\sqrt{N_xN_y}}\sum_{\boldsymbol{n}}\sigma^{\dagger}_{\boldsymbol{n}}e^{i\boldsymbol{k}\cdot\boldsymbol{r}_{\boldsymbol{n}}}$. Then, we arrive at the Hamiltonian, $H_{exc-SP}=\sum_{\boldsymbol{k}}H_{exc-SP,\boldsymbol{k}}=\sum_{\boldsymbol{k}}\mathscr{J}(\boldsymbol{k})a_{\boldsymbol{k}}\sigma^{\dagger}_{\boldsymbol{k}}+\text{h.c.}$, where

$$\mathscr{J}(\boldsymbol{k})=\sqrt{\left(\frac{N_xN_y}{S}\right)\left(\frac{\omega(\boldsymbol{k})}{2\epsilon_0 L_{\boldsymbol{k}0}}\right)}e^{-\alpha_0(\boldsymbol{k})\bar{z}(\boldsymbol{k})}\boldsymbol{\mu}\cdot\boldsymbol{E}(\boldsymbol{k}), \tag{96}$$

and $\boldsymbol{k}$ runs for all the allowed discretized wavevectors $k_i=-\frac{\pi}{\Delta_i}+\frac{2\pi}{N_i\Delta_i}q_i$ for $q_i=0,1,\cdots,N_i-1$.

Within the MFA, we have achieved to represent each nanopillar as a single collective transition dipole $\boldsymbol{\mu}_{\boldsymbol{n}}$ associated with the operator $\sigma^{\dagger}_{\boldsymbol{n}}$. There is, however, an ambiguity in this approximation, namely, the criterion to optimize the parameter $\bar{z}(\boldsymbol{k})$. We will discuss this in subsection 3 below.

2. *Beyond the MFA*

Going back to Supplementary Equation (94.1) and assuming $\boldsymbol{p}_{\boldsymbol{m}s}=\boldsymbol{p}_{\boldsymbol{n}}$, we can consider alternative nanopillar modes. We follow González-Tudela, *et al* [10]. Let us define the new modes (distinguished from the others by the overbar notation),

$$\bar{\sigma}_{\boldsymbol{n}}^{\dagger}(\boldsymbol{k}) = \frac{1}{\sqrt{\mathcal{N}}}\sum_{\boldsymbol{m}s}\boldsymbol{p}_{\boldsymbol{n}}\cdot\boldsymbol{E}(\boldsymbol{k},z_s)e^{i\boldsymbol{k}\cdot(\boldsymbol{r}_m-\boldsymbol{r}_n)-\alpha_0(\boldsymbol{k})z_s}b_{\boldsymbol{m}s}^{\dagger}, \tag{97}$$

where the corresponding normalization is given by,

$$\begin{aligned}\mathcal{N} &= \sum_{\boldsymbol{m}s}\left|\boldsymbol{p}_{\boldsymbol{n}}\cdot\boldsymbol{E}(\boldsymbol{k},z_s)e^{i\boldsymbol{k}\cdot(\boldsymbol{r}_m-\boldsymbol{r}_n)-\alpha_0(\boldsymbol{k})z_s}\right|^2\\ &\approx \frac{N_{np}}{W_z}\int_{z_0}^{z_f}dz|\boldsymbol{p}_{\boldsymbol{n}}\cdot\boldsymbol{E}(\boldsymbol{k},z)|^2e^{-2\Re\alpha_0(\boldsymbol{k})z}.\end{aligned} \tag{98}$$

where we identified $z_f - z_0 = W_z$ as the vertical thickness of each nanopillar. Introducing the corresponding $\boldsymbol{k}$ mode $\bar{\sigma}_{\boldsymbol{k}}^{\dagger} = \frac{1}{\sqrt{N_xN_y}}\sum_{\boldsymbol{n}}\bar{\sigma}_{\boldsymbol{n}}^{\dagger}(\boldsymbol{k})e^{i\boldsymbol{k}\cdot\boldsymbol{r}}$, Supplementary Equation (94.1) becomes $H_{exc-SP} = \sum_{\boldsymbol{k}}H_{exc-SP,\boldsymbol{k}} \approx \sum_{\boldsymbol{k}}\bar{\mathcal{J}}(\boldsymbol{k})a_{\boldsymbol{k}}\bar{\sigma}_{\boldsymbol{k}}^{\dagger}$+h.c., where,

$$\bar{\mathcal{J}}(\boldsymbol{k}) \approx \sqrt{\rho\left(\frac{\omega(\boldsymbol{k})}{2\epsilon_0L_{\boldsymbol{k}0}}\right)}\sqrt{\int_{z_0}^{z_f}dze^{-2\Re\alpha_0(\boldsymbol{k})z}|\boldsymbol{p}_{\boldsymbol{n}}\cdot\boldsymbol{E}(\boldsymbol{k},z)|^2}, \tag{99}$$

where we have identified

$$\rho = \frac{N_xN_yN_{np}}{SW_z} = \underbrace{\frac{(N_xW_x)(N_yW_y)}{S}}_{<1}\rho_{np} \tag{100}$$

as the *average* density of chromophores in the organic superlattice which, due to the "void space" between nanopillars, is lower than ρ_{np}. Except for a different convention in the phases of our exciton modes $\bar{\sigma}_{\boldsymbol{k}}^{\dagger}$, this solution has the same structure as the one presented in [10], even though the latter deals with an organic layer of uniform density.

3. *Comparison*

When we wrote $H_{exc-SP} \approx \sum_{\boldsymbol{k}}\mathcal{J}(\boldsymbol{k})a_{\boldsymbol{k}}\sigma_{\boldsymbol{k}}^{\dagger}$ + h.c., we made a MFA to Supplementary Equation (94.1) by invoking definitions for the mode $\sigma_{\boldsymbol{n}}^{\dagger}$ and the coupling $\mathcal{J}(\boldsymbol{k})$ (Supplementary Equations (95) and (96)). The essence of this approximation is the exponential factor $e^{-\alpha_0(\boldsymbol{k})\bar{z}(\boldsymbol{k})}$ in $\mathcal{J}(\boldsymbol{k})$, which implies that when each nanopillar interacts with the $\boldsymbol{k}$-th electromagnetic mode, it behaves as a collective dipole placed at the effective height $z = \bar{z}(\boldsymbol{k})$. On the other hand, when going beyond the MFA, we introduced $\bar{\sigma}_{\boldsymbol{n}}^{\dagger}(\boldsymbol{k})$ and $\bar{\mathcal{J}}(\boldsymbol{k})$ (Supplementary Equations (97) and (99)) and showed that $H_{exc-SP} \approx \sum_{\boldsymbol{k}}\bar{\mathcal{J}}(\boldsymbol{k})a_{\boldsymbol{k}}\bar{\sigma}_{\boldsymbol{k}}^{\dagger}$ + h.c. is an exact representation of Supplementary Equation (94.1) (notwithstanding the excellent approximations of converting the sums over chromophores to integrals and the PBCs). Hence, $\bar{\sigma}_{\boldsymbol{k}}^{\dagger}$ (and not $\sigma_{\boldsymbol{k}}^{\dagger}$) is the natural exciton mode which couples to the SP mode $a_{\boldsymbol{k}}^{\dagger}$.

The solution beyond MFA might be a more convenient description if one is interested in a careful description of the energetics of the problem. However, for purposes of the topological characterization of the plexciton band-structure, it is more pertinent to adopt the MFA description. Notice from Supplementary Equation (97) that $\bar{\sigma}_{\boldsymbol{n}}^{\dagger}(\boldsymbol{k})$ depends explicitly on $\boldsymbol{k}$, so that the Fourier modes $\bar{\sigma}_{\boldsymbol{k}}^{\dagger} = \frac{1}{\sqrt{N_xN_y}}\sum_{\boldsymbol{n}}\bar{\sigma}_{\boldsymbol{n}}^{\dagger}(\boldsymbol{k})e^{i\boldsymbol{k}\cdot\boldsymbol{r}}$ have an additional dependence on $\boldsymbol{k}$ beyond the phase factor $e^{i\boldsymbol{k}\cdot\boldsymbol{r}}$. This introduces a technicality for the numerical computation of the Chern number for each plexciton band, which we wish to avoid at present. This complication does not occur in the MFA, where $\sigma_{\boldsymbol{n}}^{\dagger}$ uniformly sums over the excitons operators $b_{\boldsymbol{m}s}^{\dagger}$ for a given nanopillar regardless of $\boldsymbol{k}$. Thus, we make a compromise: we formally use the structure of the MFA, but heuristically make the energetic approximation $\left|\mathcal{J}(\boldsymbol{k})\right| = \bar{\mathcal{J}}(\boldsymbol{k})$. Comparing Supplementary Equations (96) and (99), this identity requires that $\bar{z}(\boldsymbol{k})$ satisfies,

$$\left|\boldsymbol{p}_{\boldsymbol{n}}\cdot\boldsymbol{E}(\boldsymbol{k},\bar{z}(\boldsymbol{k}))e^{-\alpha_{org0}(\boldsymbol{k})\bar{z}(\boldsymbol{k})}\right| = \sqrt{\frac{1}{W_z}\int_{z_0}^{z_f}dz|\boldsymbol{p}_{\boldsymbol{n}}\cdot\boldsymbol{E}(\boldsymbol{k},z)|^2e^{-2\Re\alpha_{org0}(\boldsymbol{k})z}}. \tag{101}$$

This constraint has the appealing physical content of computing the mean-field effective position $\bar{z}(\boldsymbol{k})$ of the collective nanopillar dipole by averaging interaction of the SP with respect to the interval $z \in [z_i, z_f]$. Operationally, however, it is not necessarily to solve for $\bar{z}(\boldsymbol{k})$, as having the value of $\bar{\mathcal{J}}(\boldsymbol{k})$ in Supplementary Equation (99) suffices for our calculations.

To remind ourselves, when dealing with the dispersion of the organic layer alone, we have coarse grained it into an effective rectangular superlattice. However, when computing the exciton-SP coupling via Supplementary Equation (99), ρ must be taken to be the density of the original monoclinic superlattice. Supplementary Equation (100) is undetermined as N_x and N_y are artificial simulation parameters, instead, we shall write

$$\rho = f\rho_{np}, \tag{102}$$

the average density is equal to the surface coverage fraction times the original nanopillar density.

4. Representative coupling values

While the simulations for the main text use the three-layer setup, it is instructive to also consider the two-layer one. Supplementary Figures 6 and 7 plot representative exciton-SP coupling values $\bar{\mathscr{J}}(\boldsymbol{k})$ using Supplementary Equation (99) for the two-layer and three-layer setups, respectively. We display calculations for different orientations of the transition dipoles of the nanopillars, when $\boldsymbol{\mu} \parallel \hat{\boldsymbol{k}}$, $\boldsymbol{\mu} \parallel \hat{\boldsymbol{\theta}}_{\boldsymbol{k}}$, and $\boldsymbol{\mu} \parallel \hat{\boldsymbol{z}}$. Throughout the plots, we have chosen silver Drude parameters $\epsilon_\infty = 3.7$, $\omega_P = 8.8$ eV and $g = 0.3$. Each of the panels displays results corresponding to a particular dielectric permittivity ϵ_d and the base height of the nanopillars z_0, and fixing the same organic layer height $W_z = 70$ nm. Taking the parameters for the organic superlattice described in section A of this Supplementary Note and assuming $\rho_{np} = 38$ chromophores/nm^3, the average density using $f = 0.78$ and Supplementary Equation (102) is computed to be $\rho = 29.7$ chromophores/nm^3. Supplementary Figure 7 assumes that the MO dielectric layer has a width a and the base height of the nanopillars is also a. Supplementary Figure 6 corresponds to the potential scenario where the organic nanopillars are “embedded” in the MO dielectric layer starting at the base height z_0.

As a reminder, in the absence of the MO effect, the electric field of the $\boldsymbol{k}$-th SP mode has no tangential component (see Supplementary Equations (40.1), (72.1)). Thus, the blue curves in the plots ($\boldsymbol{\mu} \parallel \hat{\boldsymbol{\theta}}_{\boldsymbol{k}}$) must vanish identically for $g = 0$. For $g \neq 0$, these couplings scale linearly with g (see Supplementary Equations (41.1), (76)), so it is easy to predict these perturbative exciton-SP couplings for other values of g. On the other hand, the red ($\boldsymbol{\mu} \parallel \hat{\boldsymbol{k}}$) and black ($\boldsymbol{\mu} \parallel \hat{\boldsymbol{z}}$) curves are independent of the value of g, as they are $O(g^0)$. Notice that all the curves peak at short wavevectors. For the plexciton dispersion calculation in the main text, we optimized large topological anticrossing gaps by choosing the effective site energy $\bar{\omega}_{eff}$ for excitons (see Supplementary Equation (87)) such that the exciton dispersion $H_{exc,\boldsymbol{k}}$ and the SP dispersion $H_{SP,\boldsymbol{k}}$ become degenerate (Dirac points) at wavevectors $\boldsymbol{k}^*$ where the coupling to the tangential MO electric field is largest; in other words, we maximized $\bar{\mathscr{J}}(\boldsymbol{k}^*)$ for $\boldsymbol{\mu} \parallel \hat{\boldsymbol{\theta}}_{\boldsymbol{k}}$.

The simulations displayed in the main text correspond to the first (upper left corner) panel of Supplementary Figure 7, yielding $\bar{\mathscr{J}}(1.60 \times 10^7\,\mathrm{m}^{-1}) = 0.115$ eV for $\boldsymbol{\mu} \parallel \hat{\boldsymbol{\theta}}_{\boldsymbol{k}}$, or equivalently, a topological anticrossing gap of $2\bar{\mathscr{J}}(\boldsymbol{k}^*) = 0.23$ eV. This value becomes slightly larger than the linewidths of the exciton and SP modes. Note that quite often, the largest couplings occur when $\boldsymbol{\mu} \parallel \hat{\boldsymbol{z}}$, reaching values which are comparable to the exciton site energies. This regime, known as ultra-strong coupling [21], is interesting in its own right and gives rise to novel effects, which are beyond the scope of our work. Unfortunately, for our purposes, we cannot exploit these large couplings, as they do not vanish for any $\boldsymbol{k}$ and hence, does not yield Dirac points (see main text).

A few interesting trends can be obtained from scanning through ϵ_d and z_0; some of these results are displayed in Supplementary Figures 6 and 7. First, couplings $\bar{\mathscr{J}}(\boldsymbol{k})$ for $\boldsymbol{\mu} \parallel \hat{\boldsymbol{\theta}}_{\boldsymbol{k}}$ and $\boldsymbol{\mu} \parallel \hat{\boldsymbol{z}}$ decrease as z_0 increases, eventually becoming very small for photonic distances (Supplementary Figure 6h and 7h). This is not surprising, as owing to the evanescent nature of the SP fields, these couplings should be strongest for the chromophores that are closest to the interface at $z = 0$. For the three-layer setup, however, the MO effect becomes naturally very small as the MO spacer thickness a vanishes. Contrary to the evanescent decay of the field, Faraday rotation increases with propagation distance. Hence, we expect the largest MO effect in plasmons to peak at a particular MO spacer thickness a, large enough to experience Faraday rotation, but short enough that it has substantial amplitude despite of its evanescent decay. Keeping all other parameters of the simulation in the main text equal, we find that $a = 175$ nm maximizes $\bar{\mathscr{J}}(\boldsymbol{k}^*)$ at $\bar{\mathscr{J}}(1.24 \times 10^7\,\mathrm{m}^{-1}) = 0.124$ eV for $\boldsymbol{\mu} \parallel \hat{\boldsymbol{\theta}}_{\boldsymbol{k}}$. In our simulations, we do not choose this value of a because the latter is barely larger than what we get with $a = 80$ nm but, due to the exponential decay of the field as a function of distance, renders much smaller values of $\bar{\mathscr{J}}(\boldsymbol{k})$ for $\boldsymbol{\mu} \parallel \hat{\boldsymbol{k}}$ ($\bar{\mathscr{J}}(\boldsymbol{k}) < 0.25$ eV) compared with the preferred scenario ($\bar{\mathscr{J}}(\boldsymbol{k}) < 0.55$ eV). Large couplings $\bar{\mathscr{J}}(\boldsymbol{k})$ for $\boldsymbol{\mu} \parallel \hat{\boldsymbol{k}}$ guarantee a global topological gap. also these values of which are small not optimal to generate the global topological gap.

Another interesting trend is that couplings decrease as the permittivity ϵ_d increases. This is an effect of index mismatch between the different layers. A possibility to ameliorate this problem is to embed the MO material inside of a low dielectric polymer, or consider multilayer structures which adiabatically change the dielectric function as a function of height. Notice that $\bar{\mathscr{J}}(\boldsymbol{k})$ for $\boldsymbol{\mu} \parallel \hat{\boldsymbol{\theta}}_{\boldsymbol{k}}$ for the two-layer setup are about an order of magnitude larger than the corresponding couplings in the three-layer setup. This is explained by the fact that in this setup, the organic layer is embedded in the MO layer, so the MO effect does not end sharply at $z_0 = a$, as in the three-layer setup. Just to get an intuition about the plexciton dispersion that one would obtain under this hypothetical scenario, we present it in Supplementary Figure 8 with all the other

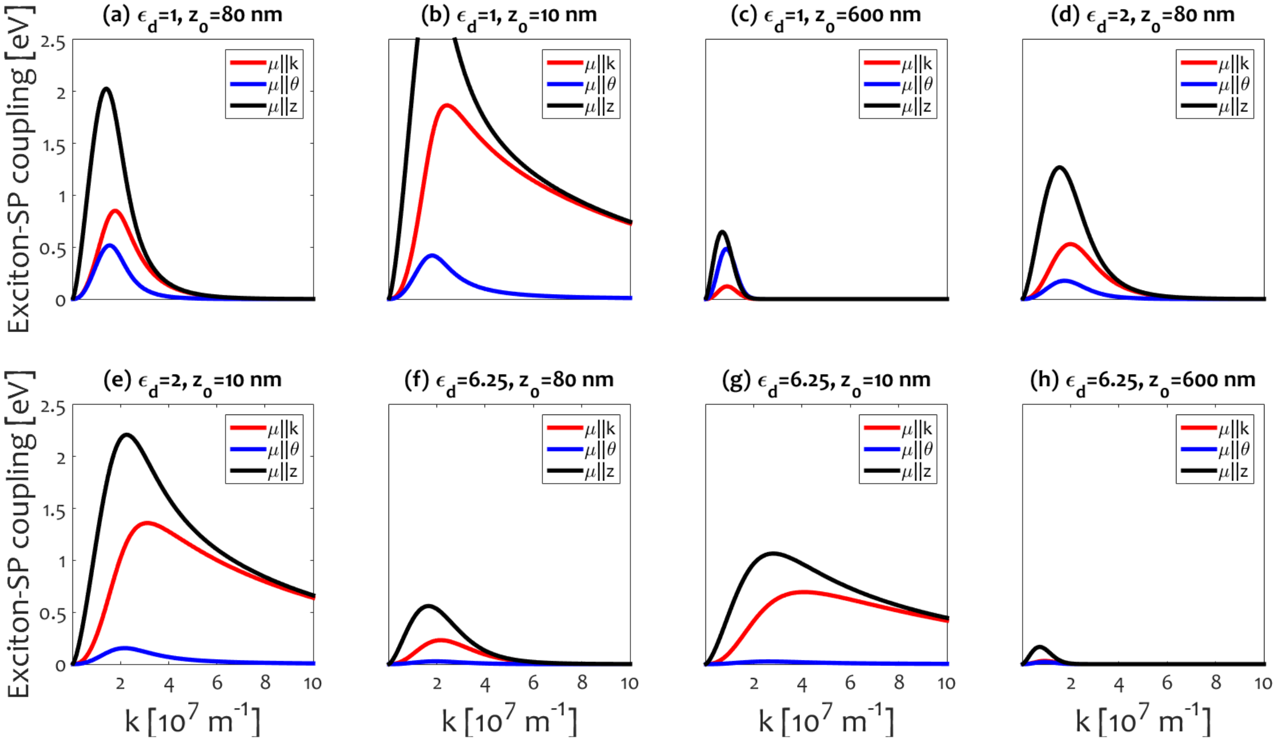

Supplementary Figure 6. *Representative exciton-SP coupling values for two-layer (metal-MO dielectric) setup.* The calculations have been carried out using Supplementary Equation (99). We display results for different orientations of the transition dipoles of the nanopillars, when $\boldsymbol{\mu} \parallel \hat{\boldsymbol{k}}$, $\boldsymbol{\mu} \parallel \hat{\boldsymbol{\theta}}$, and $\boldsymbol{\mu} \parallel \hat{\boldsymbol{z}}$. Notice that all the curves have maxima at short wavevectors. The calculations assume that $\epsilon_\infty = 3.8$, $\omega_P = 8.8$ eV, $g = 0.3$, and the height of the nanopillars being $W_z = 70$ nm.

parameters being equal to the three-layer simulation in the main text (i.e., using the coupling values in Supplementary Figure 6a). Notice that due to the large values of $\bar{\mathscr{J}}(\boldsymbol{k})$ for $\boldsymbol{\mu} \parallel \hat{\boldsymbol{\theta}}_{\boldsymbol{k}}$, all the edge states clearly reside in the global topological gap, in contrast with the edge states in the dispersion of Supplementary Figure 3b in the main text, where only a fraction is in the global topological gap. This indicates that in order to optimize the topological plexciton structures, we need to find materials providing substantial $\bar{\mathscr{J}}(\boldsymbol{k}^*)$ values (> 0.1 eV) for $\boldsymbol{\mu} \parallel \hat{\boldsymbol{\theta}}_{\boldsymbol{k}}$ we need to find materials with large g values at the UV/visible (recall the crossing between the SP and exciton dispersions at $\boldsymbol{k}^*$ happens at 2.86 eV in our calculation). Some examples of the latter are Co alloy films [22], orthoferrites [23], or spinels [24]. A caveat about the latter is that they are also highly absorptive at those same wavelengths (large imaginary part of ϵ_d, which we have neglected in this work). Ce substituted YIG has less of a problem in that regard [25]. Typical solutions to weak MO effects rely on the formation of Fabry-Perot cavities which force propagating modes to bounce many times within an MO material [26]. A plasmonic analogue of this effect is desirable for the present problem. We are currently working towards these directions in order to induce strong MO effects.

5. *Bulk energy dispersion cross-sections*

For illustration, we display dispersion relations for the three-layer setup (which is ultimately the calculation we display in the main text) in order to ellucidate the onset of the formation of Dirac points and their opening due to the MO effect.

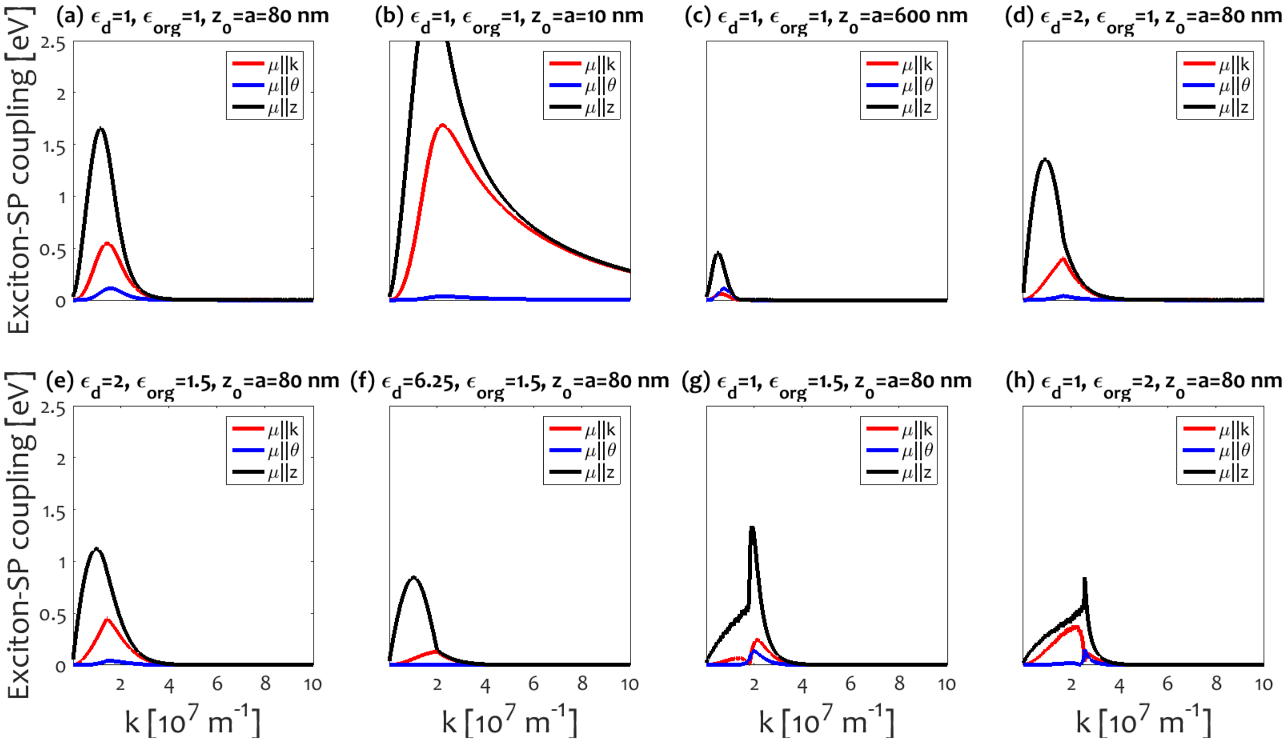

Supplementary Figure 7. *Representative exciton-SP coupling values for three-layer (metal-MO dielectric-organic) setup.* Just as with Supplementary Figure 6, the calculations have been carried out using Supplementary Equation (99). We display results for different orientations of the transition dipoles of the nanopillars, when $\boldsymbol{\mu} \parallel \hat{\boldsymbol{k}}$, $\boldsymbol{\mu} \parallel \hat{\boldsymbol{\theta}}$, and $\boldsymbol{\mu} \parallel \hat{\boldsymbol{z}}$. Notice that all the curves have maxima at short wavevectors. The calculations assume that $\epsilon_\infty = 3.8$, $\omega_P = 8.8$ eV, $g = 0.3$, and the height of the nanopillars being $W_z = 70\,$nm.

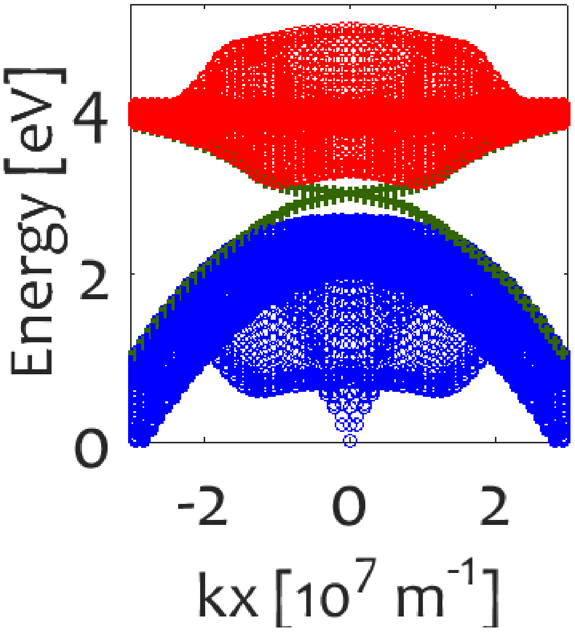

Supplementary Figure 8. *Plexciton dispersion for two-layer setup.* All parameters are held equal to those used in Supplementary Figure 3b in the main text, except for using a two-layer setup rather than a three-layer one. Notice that all the topological edge states reside in the global gap, which is possible due to the increase MO effect with respect to the three-layer simulation.

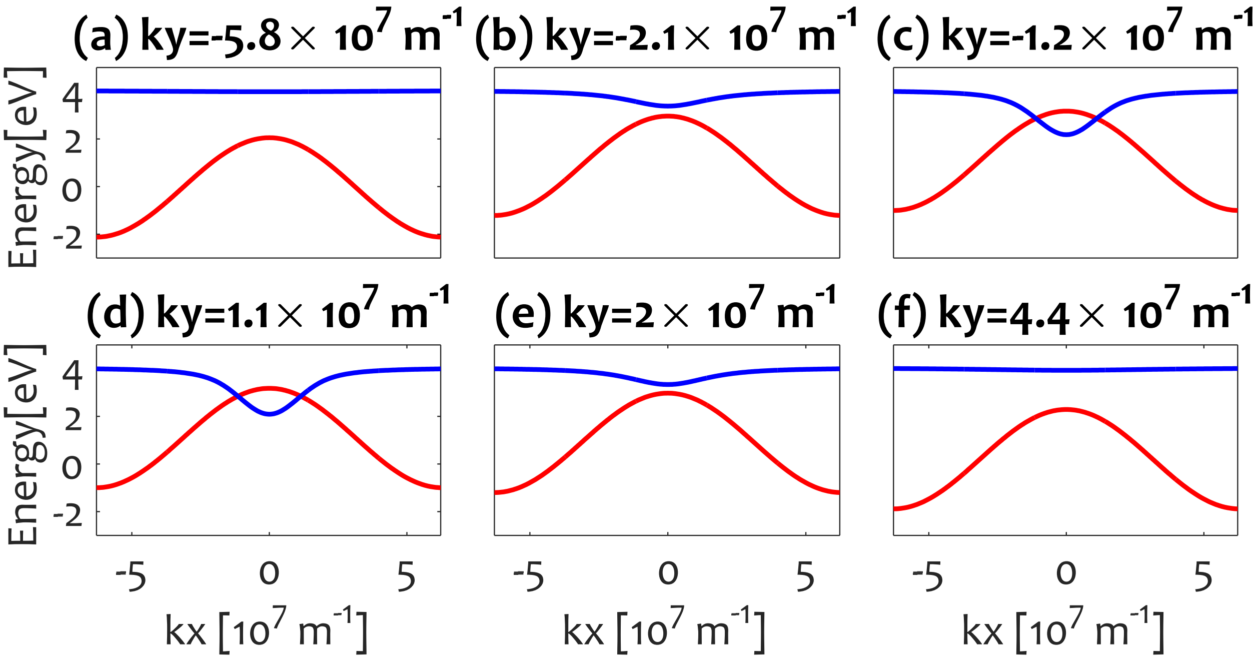

Supplementary Figure 9. *Cross-sections of the independent bulk dispersion curves of exciton (red) and plasmon (blue) modes for fixed values of k_y.* The calculations assume that $\epsilon_\infty = 3.8$, $\omega_P = 8.8$ eV, the height of the nanopillars is $W_z = 70$ nm. The plotted energies are independent of g in our perturbation theory framework.

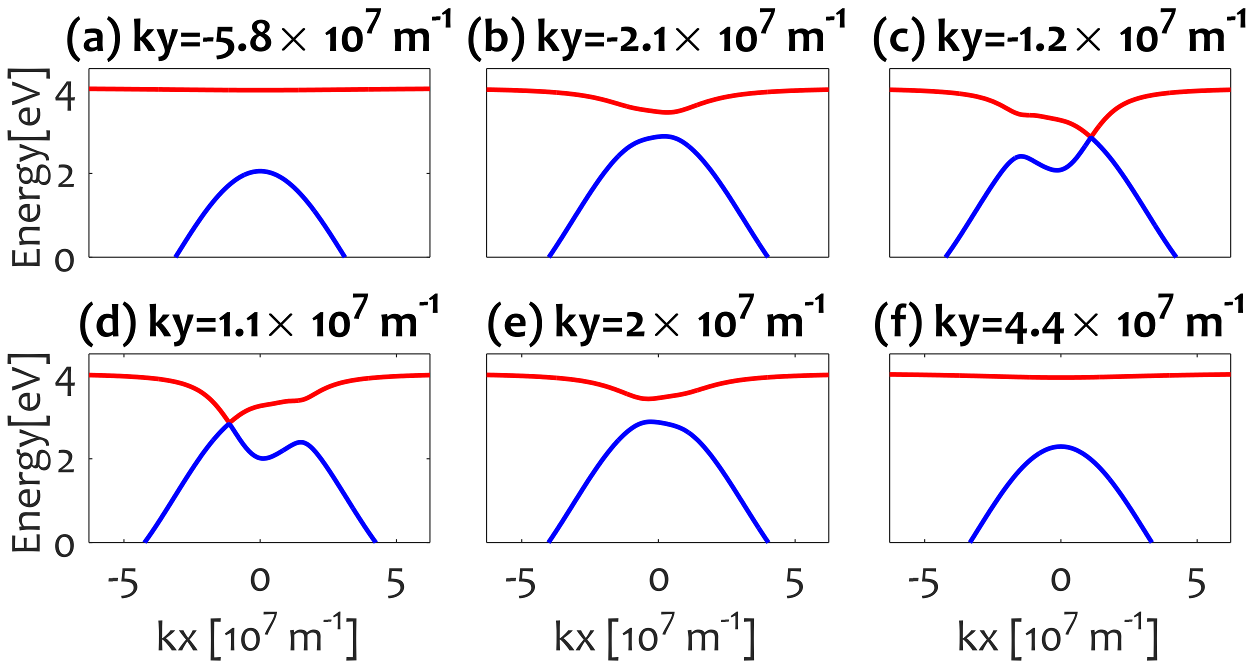

Supplementary Figure 10. *Cross-sections of the bulk plexciton dispersion curves for fixed values of k_y and g=0.* The rest of the parameters remains as in Supplementary Figure (9). The upper (red) and lower (blue) plexcitons emerging from the coupling of modes in Supplementary Figure (9) in the absence of the MO effect. Notice that Dirac points emerge in panels (c) and (d), corresponding to the different wavevectors where the coupling between the modes vanishes.

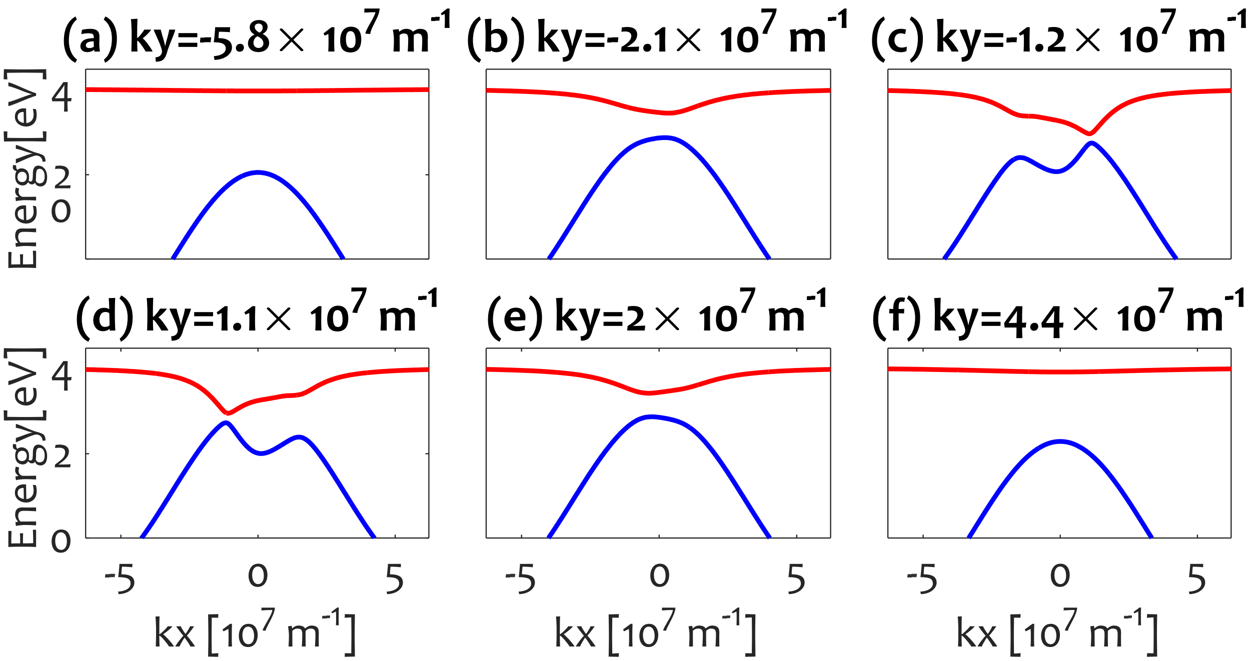

Supplementary Figure 11. *Cross-sections of the bulk plexciton dispersion curves for fixed values of* k_y *and g=0.3.* The rest of the parameters remains as in Supplementary Figure (9). The upper (red) and lower (blue) plexcitons emerging from the coupling of modes in Supplementary Figure (9) in the presence of the MO effect. Notice that Dirac points in panels (c) and (d) of Supplementary Figure (10) do not survive in this setup due to an $O(g)$ perturbation. For a calculation with open-boundary conditions, this gap is populated with topologically protected edge states (see main text, Supplementary Figure 3).

[1] Kuzmiak, V., Eyderman, S. & Vanwolleghem, M. Controlling surface plasmon polaritons by a static and/or time-dependent external magnetic field. *Phys. Rev. B* **86**, 045403 (2012).

[2] Onbasli, M., Goto, T., Sun, X., Huynh, N. & Ross, C. A. Integration of bulk-quality thin film magneto-optical cerium-doped yttrium iron garnet on silicon nitride photonic substrates. *Opt. Express* **22**, 25183–25192 (2014).

[3] Dionne, G. F. *Magnetic oxides* (Springer, 2009).

[4] West, P. *et al.* Searching for better plasmonic materials. *Laser Photon. Rev.* **4**, 795–808 (2010).

[5] Bellessa, J., Bonnand, C., Plenet, J. C. & Mugnier, J. Strong coupling between surface plasmons and excitons in an organic semiconductor. *Phys. Rev. Lett.* **93**, 036404 (2004).

[6] Govorov, A. O. *et al.* Exciton-plasmon interaction and hybrid excitons in semiconductor-metal nanoparticle assemblies. *Nano Lett.* **6**, 984–994 (2006).

[7] Sugawara, Y., Kelf, T. A., Baumberg, J. J., Abdelsalam, M. E. & Bartlett, P. N. Strong coupling between localized plasmons and organic excitons in metal nanovoids. *Phys. Rev. Lett.* **97**, 266808 (2006).

[8] Chang, D. E., Sørensen, A. S., Hemmer, P. R. & Lukin, M. D. Strong coupling of single emitters to surface plasmons. *Phys. Rev. B* **76**, 035420 (2007).

[9] Salomon, A., Gordon, R. J., Prior, Y., Seideman, T. & Sukharev, M. Strong coupling between molecular excited states and surface plasmon modes of a slit array in a thin metal film. *Phys. Rev. Lett.* **109**, 073002 (2012).

[10] González-Tudela, A., Huidobro, P. A., Martín-Moreno, L., Tejedor, C. & García-Vidal, F. J. Theory of strong coupling between quantum emitters and propagating surface plasmons. *Phys. Rev. Lett.* **110**, 126801 (2013).

[11] Törmä, P. & Barnes, W. Strong coupling between surface plasmon polaritons and emitters: a review. *Rep. Prog. Phys.* **78**, 013901 (2015).

[12] Fan, H. *et al.* Self-assembled aerogel-like low dielectric constant films. *J. Non-Cryst. Solids* **285**, 79–83 (2001).

[13] Chiu, K. & Quinn, J. Magneto-plasma surface waves in solids. *Nuovo Cimento B* **10**, 1–20 (1972).

[14] Maier, S. A. *Plasmonics: Fundamentals and Applications* (Springer, 2007).

[15] Novotny, L. & Hecht, B. *Principles of Nano-Optics* (Cambridge University Press, 2012).

[16] Archambault, A., Marquier, F., Greffet, J.-J. & Arnold, C. Quantum theory of spontaneous and stimulated emission of surface plasmons. *Phys. Rev. B* **82**, 035411 (2010).

[17] Fidder, H., Knoester, J. & Wiersma, D. A. Optical properties of disordered molecular aggregates: A numerical study. *J. Chem. Phys.* **95**, 7880–7890 (1991).

[18] DeVoe, H. Optical properties of molecular aggregates. i. classical model of electronic absorption and refraction. *J. Chem. Phys.* **41**, 393–400 (1964).

[19] DeVoe, H. Optical properties of molecular aggregates. ii. classical theory of the refraction, absorption, and optical activity of solutions and crystals. *J. Chem. Phys.* **43**, 3199–3208 (1965).

[20] Valleau, S. *et al.* Electromagnetic study of the chlorosome antenna complex of chlorobium tepidum. *ACS Nano* **8**, 3884–3894 (2014).

[21] Todorov, Y. *et al.* Ultrastrong light-matter coupling regime with polariton dots. *Phys. Rev. Lett.* **105**, 196402 (2010).

[22] Temnov, V. *et al.* Active magneto-plasmonics in hybrid metal–ferromagnet structures. *Nat. Photon.* **4**, 107–111 (2010).

[23] Kahn, F. J., Pershan, P. S. & Remeika, J. P. Ultraviolet magneto-optical properties of single-crystal orthoferrites, garnets, and other ferric oxide compounds. *Phys. Rev.* **186**, 891–918 (1969).

[24] Fontijn, W. F. J., Van der Zaag, P. J., Feiner, L. F., Metselaar, R. & Devillers, M. A. C. A consistent interpretation of the magneto-optical spectra of spinel type ferrites. *J. Appl. Phys.* **85**, 5100 (1999).

[25] Sun, X. Y. *et al.* Single-step deposition of cerium-substituted yttrium iron garnet for monolithic on-chip optical isolation. *ACS Photonics* **2**, 856–863 (2015).

[26] Inoue, M. & Fujii, T. A theoretical analysis of magneto-optical faraday effect of yig films with random multilayer structures. *J. App. Phys.* **81**, 5659–5661 (1997).
